# Development of an assessment method for freely moving nonhuman primates’ eating behavior using manual and deep learning analysis

**DOI:** 10.1016/j.heliyon.2024.e25561

**Published:** 2024-02-05

**Authors:** Leslie Jaesun Ha, Meelim Kim, Hyeon-Gu Yeo, Inhyeok Baek, Keonwoo Kim, Miwoo Lee, Youngjeon Lee, Hyung Jin Choi

**Affiliations:** a Department of Biomedical Sciences, Wide River Institute of Immunology, Neuroscience Research Institute, Seoul National University College of Medicine, Republic of Korea; b National Primate Research Center, Korea Research Institute of Bioscience and Biotechnology (KRIBB), Republic of Korea; c KRIBB School of Bioscience, Korea National University of Science and Technology, Republic of Korea; d School of Life Sciences, BK21 Plus KNU Creative BioResearch Group, Kyungpook National University, Republic of Korea; e Department of Preventive Medicine, Yonsei University College of Medicine, Seoul, Republic of Korea; f Center for Wireless and Population Health Systems (CWPHS), University of California, San Diego, La Jolla, CA, 92093, USA; g Herbert Wertheim School of Public Health and Human Longevity Science, University of California San Diego, San Diego, CA, United States

**Keywords:** Non-human primate, Eating behaviors, Hunger, Palatability, Assessment method, Deep learning-based analysis

## Abstract

**Purpose:**

Although eating is imperative for survival, few comprehensive methods have been developed to assess freely moving nonhuman primates' eating behavior. In the current study, we distinguished eating behavior into appetitive and consummatory phases and developed nine indices to study them using manual and deep learning-based (DeepLabCut) techniques.

**Method:**

The indices were utilized to three rhesus macaques by different palatability and hunger levels to validate their utility. To execute the experiment, we designed the eating behavior cage and manufactured the artificial food. The total number of trials was 3, with 1 trial conducted using natural food and 2 trials using artificial food.

**Result:**

As a result, the indices of highest utility for hunger effect were approach frequency and consummatory duration. Appetitive composite score and consummatory duration showed the highest utility for palatability effect. To elucidate the effects of hunger and palatability, we developed 2D visualization plots based on manual indices. These 2D visualization methods could intuitively depict the palatability perception and hunger internal state. Furthermore, the developed deep learning-based analysis proved accurate and comparable with manual analysis. When comparing the time required for analysis, deep learning-based analysis was 24-times faster than manual analysis. Moreover, temporal and spatial dynamics were visualized via manual and deep learning-based analysis. Based on temporal dynamics analysis, the patterns were classified into four categories: early decline, steady decline, mid-peak with early incline, and late decline. Heatmap of spatial dynamics and trajectory-related visualization could elucidate a consumption posture and a higher spatial occupancy of food zone in hunger and with palatable food.

**Discussion:**

Collectively, this study describes a newly developed and validated multi-phase method for assessing freely moving nonhuman primate eating behavior using manual and deep learning-based analyses. These effective tools will prove valuable in food reward (palatability effect) and homeostasis (hunger effect) research.

## Graphical abstract

**Figure:**
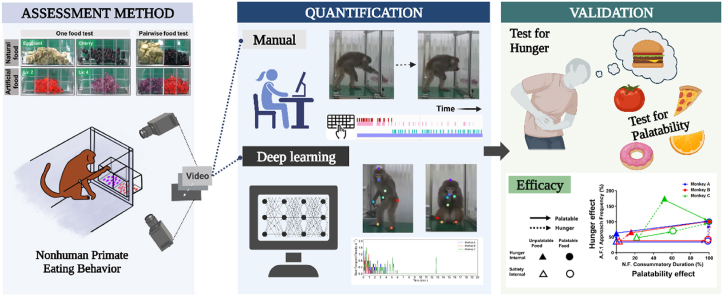

## 1 Introduction

Given that eating is a requisite for survival, defining and quantifying multiple aspects of eating behavior is a critical component of eating-related research. Eating behavior comprises multi-phase behaviors that are initiated with an appetitive phase (search and approach) that sequentially leads to a consummatory phase (biting, chewing, and ingesting when the animal is proximate to the food) [1]. Since seeking and consummatory behaviors have distinct characteristics regarding the motivational state and behavioral decision, distinct functional populations guide each phase of behavior. Recent studies have shown that seeking and consummatory behaviors are regulated by distinct neural populations related to aggression and mating [[2], [3], [4]]. Therefore, to investigate the appetitive and consummatory phases of eating behavior, efficient assessment methods are required. Although various studies have reported on the conceptual phases and assessment methods for the eating behaviors of rodents, nonhuman primates, and humans [5], effective and easily applicable assessment methods for the appetitive and consummatory eating phases of freely moving nonhuman primates are lacking.

Recently, multiple eating behavior assessment models have been developed and assessed, resulting in numerous research methods capable of assessing eating behavior in humans, including the Dutch Eating Behavior Questionnaire [6], Yale Food Addiction Scale [7,8], and Appetite Visual Analogue Scale [[9], [10], [11]]. Moreover, several studies have assessed the general eating behavior (e.g., social interaction [12,13], eating behavior in wild [[14], [15], [16]], eating behavior in relation to brain processes [[17], [18], [19], [20]], and behavior change [21]), food motivation (e.g., reaction time [22] and secondary reinforcement of reward [23]), and consummatory behaviors (e.g., measuring food intake and eating time [15,24], or ingestive behavior [14]) of nonhuman primates. However, few quantitative assessment methods are available for freely moving eating behavior with diverse indices.

Hunger and palatability are the major drivers of eating behavior [25]. In fact, several studies have utilized hunger in nonhuman primates to investigate economic preference [26], olfactory and visual representation [27], hypothalamic circuitry [28,29], and correlation with other brain areas [30,31]. When considering palatability, various studies utilizing high-fat or high-sugar foods have provided valuable information regarding how palatability impacts changes in internal states [[32], [33], [34], [35], [36]]. Therefore, hunger and palatability are useful components for validating the efficacy and utility of tools to investigate eating behavior.

Recently, many animal studies have been published based on deep learning, which automatically assesses the markerless position and pose estimation of user-defined body parts [[37], [38], [39], [40], [41]]. Several primate studies have also used deep learning-based analysis methods such as MacaquePose [42], OpenPose [43], OpenMonkeyStudio [44,45], and DeepLabCut [38,[46], [47], [48]] for arm movements with head-fixed [[46], [47], [48], [49]] and social-related poses [42]. Meanwhile, there is a current dearth of studies comparing manual and deep learning with similar behavior indices.

The purpose of this study is to develop an assessment method for the appetitive and consummatory eating phases of nonhuman primates. Moreover, we aim to compare the performance and labor time requirement between manual and deep learning-based analysis. To validate their utility, the developed methods are subsequently tested based on internal state (hunger internal state, neutral internal state (neither sated nor hungry), and satiety internal state) and palatability (palatable food and unpalatable food). Taken together, the findings of this study provide methodological insights into nonhuman primate ethology. The outline of our method is as follows; using three female macaques, designed cage, conditioning test, different degree of hunger and palatability, one food test, pairwise food test, natural food, artificial food, feeding behavior assessment indices, manual and deep learning-based analysis.

## 2 Results

### 2.1 Experimental scheme and behavior analysis method for manual and deep learning-based analyses

**Fig. 1:**
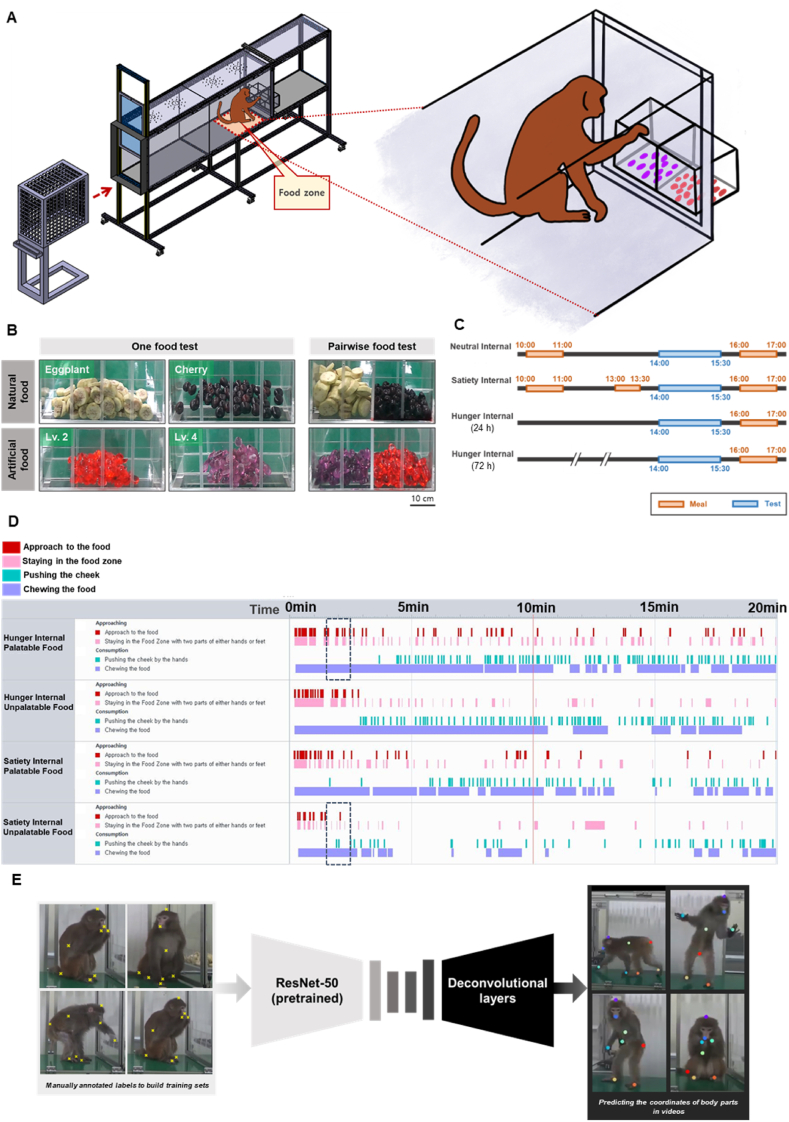
Experimental scheme and behavior analysis method for manual and deep learning-based analyses. **(A)** Scheme of experimental setting. The designed experimental cage with the food zone (red dotted line) and single-primate transfer cage docking on the tunnel entrance using a sliding door. **(B)** One-food test tray and pairwise-food test tray with the natural and artificial food stimuli (palatable food, unpalatable food) **(C)** Experimental time schedule of neutral internal state, satiety internal state, and hunger internal state; one-food test for 72 h and pairwise-food test for 24 h. **(D)** Manual analysis using Observer XT. Visualization of each index for event and duration. The black dotted square timeline is reflected in Movie S1 and S2. **(E)** DeepLabCut was used to label and predict the coordinates of each body part for the deep learning-based analysis.

We used three macaques in an experiment with a food zone in an experimental cage (Fig. 1A). To investigate the palatability effect, we conducted with palatable food and unpalatable food in both artificial (Fig. S1) and natural food conditions. Further, one food and pairwise food test was executed (Fig. 1B). To assess the hunger effect, different degrees of internal states were set (Fig. 1C). Behavioral analysis was conducted through manual (Fig. 1D) and deep learning-based (Fig. 1E) methods.

### 2.2 The classification of eating behavior phase and behavior indices for manual and deep learning-based analyses

Manual and deep learning-based analyses of eating behavior were divided into two different phases: appetitive and consummatory. While the behaviors in the appetitive phase were related to food, they only included approaching the tray and food without actually consuming it. The behaviors in the consummatory phase are defined as behaviors related to food consumption (Table 1). Behavior indices were defined by abbreviations of code. All abbreviations are made by taking the first letter of measured behavior. ‘M’ and ‘D’ stand for manual and deep learning-based analysis, respectively.

**Table 1:** **The classification of eating behavior phase and behavior indices**. The eating behavior indices of manual and deep learning-based analyses were divided into two different phases: appetitive and consummatory. Behavior indices were defined by abbreviations of code. Appetitive phase: frequency of food and tray approach (Approach Frequency; M-AF/D-AF), duration in the food zone (Appetitive Duration; M-AD/D-AD), latency to first approach (Approach Latency; M-AL), and score for appetitive volition (Appetitive Composite score; M-AC). Consummatory Phase: frequency of cheek pushing (Cheek pushing Frequency; M − CF), duration of food chewing (Consummatory Duration; M-CD) and frequency of bout (Consummatory Frequency; D-CF). All abbreviations are made by taking the first letter of measured behavior. ‘M’ and ‘D’ stand for manual and deep learning-based analysis, respectively.

Conceptual Phases	Measured Behavior	Index	Code
**Appetitive**	Approach Frequency	Number (n)	**M-AF/D-AF**
	Appetitive Duration	Duration (s)	**M-AD/D-AD**
	Approach Latency	Latency (s)	**M-AL**
	Appetitive Composite score	AF/AL (a.u.)	**M-AC**
**Consummatory**	Cheek pushing Frequency	Number (n)	**M-CF**
	Consummatory Frequency	Number (n)	**D-CF**
	Consummatory Duration	Duration (s)	**M-CD**

For the appetitive phase, four behavior measures were developed using manual analysis: frequency of food approach (M-AF, Approach Frequency); duration in food zone (M-AD, Appetitive Duration), latency to first approach (M-AL, Approach Latency), the appetitive volition score (M-AC, Appetitive Composite score) which was computed by AF/AL ratio. We also developed deep learning-based indices for the one-food test. Analogous to the manual analysis, the deep learning-based analysis consists of appetitive and consummatory phases. Two indices were applied for the appetitive phase: frequency of tray approach (D-AF, Approach Frequency) and duration in food zone (D-AD, Appetitive Duration). For the consummatory phase, two behavior measures were developed using manual analysis: frequency of cheek pushing (M − CF, Cheek pushing Frequency) and duration of food chewing (M-CD, Consummatory Duration). For the deep learning-based analysis, the frequency of bout (D-CF, Consummatory Frequency) was the only index applied.

### 2.3 The performance of the developed eating behavior indices validating the effects of hunger and palatability

**Fig. 2:**
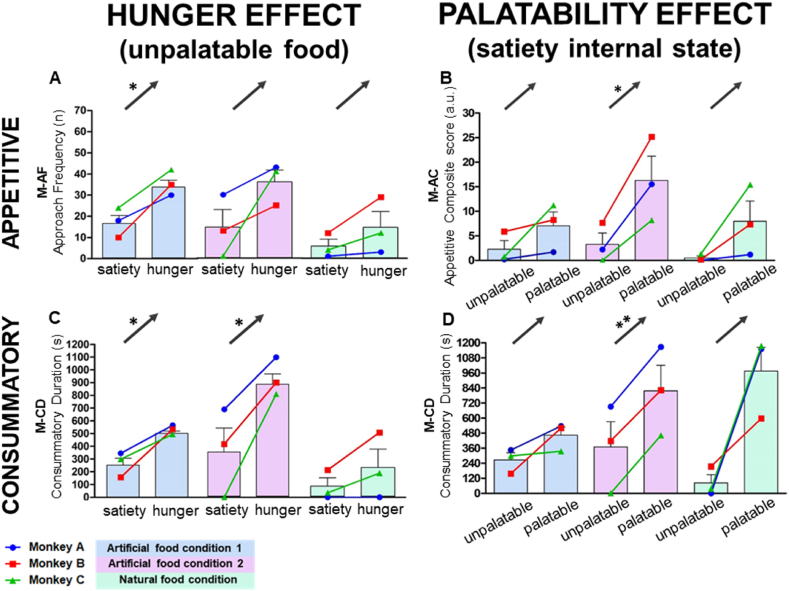
The performance of the developed eating behavior indices validating the effects of hunger and palatability. Manual analyses indices for appetitive and consummatory phase. **(A)** Approach frequency on hunger internal state was consistently higher than satiety internal state (using unpalatable food, hunger effect) (artificial food condition 1, *P* = 0.0395 (*)). **(B)** Appetitive composite score using palatable food was consistently higher than unpalatable food stimuli (with satiety internal state, palatability effect) (artificial food condition 2, *P* = 0.0409 (*)). **(C)** Consummatory duration on hunger internal state was higher than satiety internal state in artificial food conditions (using unpalatable food, hunger effect) (artificial food condition 1, *P* = 0.0434 (*), artificial food condition 2, *P* = 0.0438 (*)). **(D)** Consummatory duration using palatable food was consistently higher than unpalatable food stimuli (with satiety internal state, palatability effect) (artificial food condition 2, *P* = 0.0025 (**)). The black arrows on the graph indicate that all the monkeys have a same direction (upward or downward) partially along with the *p-value*; *P* < 0.05 (*) and *P* < 0.01 (**). The absolute *P* value is shown on Table S2.

We tested the developed methods to validate their utility for measuring hunger (hunger internal state vs. satiety internal state) and palatability (palatable food vs. unpalatable food) effects. Total three replicated tests were performed. The natural food stimuli test was performed once (natural food condition). In addition, the artificial food stimuli test was repeated twice (artificial food conditions 1 and 2) since experiments on artificial foods are manufactured with a strictly controlled ingredient. Among the nine developed indices, those with the best ability to reflect the hunger and palatability effects were determined (Table S1). To this end, all food stimuli and internal states (36 results: nine indices with four conditions) of appetitive and consummatory phases were assessed. To identify the indices with the best performance, we quantified the performance score of the index based on statistical significance (p-value; asterisk) and identical direction of all three monkeys (altogether; black arrow). Therefore, the best score was achieved with unpalatable food stimuli in the hunger effect and satiety internal state in the palatable effect (representative indices in Table S1). Among the appetitive phase, M-AF and the M-AC score demonstrated the highest performance for assessing hunger and palatable effects, respectively. Further, among the consummatory phase, M-CD demonstrated the highest performance for analyzing hunger and palatable effects (Fig. 2).

Regarding the hunger effect within the appetitive phase on unpalatable food stimuli, the M-AF was “altogether” (all three monkeys showing identical directions) higher in the hunger internal state than in the satiety internal state during artificial food condition 2 and the natural food condition and was significantly (artificial food condition 1, *P* = 0.0395 (*)) higher in the hunger internal state during artificial food condition 1 (Fig. 2A). For the consummatory phase, the M-CD was significantly (artificial food condition 1, *P* = 0.0434 (*), artificial food condition 2, *P* = 0.0438 (*)) higher in the hunger internal state than in the satiety internal state during both artificial food conditions (Fig. 2C). The remaining behavior indices for the hunger effect, excluding the representative, are presented in Fig. S3 under the palatable food and unpalatable food stimuli. Additionally, when applied to Table S1, the representative indices scored >4 points, while all other indices scored <4 points (Fig. S3).

Regarding the palatability effect within the appetitive phase on the satiety internal state, M-AC was “altogether” higher with palatable food stimuli than with unpalatable food stimuli in artificial food condition 1 and the natural food condition and was significantly (artificial food condition 2, *P* = 0.0409 (*)) higher in artificial food condition 2 (Fig. 2B). For the consummatory phase M-CD, an “altogether” longer duration was observed with palatable food stimuli than unpalatable food stimuli during artificial food condition 1 and the natural food condition and was significantly (artificial food condition 2, *P* = 0.0025 (**)) longer with palatable food stimuli during artificial food condition 2 (Fig. 2D). The remaining behavior indices for the palatability effect are presented in Fig. S4 under the hunger internal and satiety internal states. Similar to the hunger effect, when applied to Table S1, the representative indices scored >4 points, while all other indices scored <4 points (Fig. S4).

Taken together, to investigate the hunger effect, application of AF is effective for the appetitive phase and M-CD is effective for the consummatory phase with unpalatable food stimuli. In addition, for the palatability effect, M-AC for the appetitive phase and M-CD for the consummatory phase are effective with a satiety internal state. Along with these representative results, as demonstrated by the results in Table S1, it can be inferred that the impact of unpalatable food on the hunger effect is greater, as evidenced by the higher total score for unpalatable food compared to palatable food. Similarly, in palatability, the total score for the satiety internal state was higher than that for the hunger internal state.

No statistically significant result was obtained when using the Bonferroni correction method and the Wilcoxon signed-rank test.

### 2.4 Pairwise-food test for verification of food preference applied to manual eating behavior indices

To assess food preference according to palatability, we conducted a pairwise-food test. In the artificial food condition, M-AF was significantly higher with palatable food stimuli than with unpalatable food stimuli during the neutral internal and satiety internal states (Fig. S5A, neutral internal state: *P* = 0.0031 (**), satiety internal state: *P* = 0.0122 (*)). M-AL with palatable food stimuli had low latency, while one or two monkeys did not make contact with unpalatable food stimuli (Fig. S5B). Dropping the food (M-AF-d and M-AL-d) was deemed to be ambiguous behavior as it might occur unintentionally (Movie S4, Figs. S5C and S5D). The CA was significantly higher with palatable food stimuli than with unpalatable food stimuli during the hunger, neutral, and especially satiety internal states (Fig. S5E, Movie S3).

In the natural food condition, M-AF was “altogether” higher with palatable food stimuli than with unpalatable food stimuli only in the satiety internal state (Fig. S5F). The M-AL, like the artificial food condition, showed that there was no contact with unpalatable food stimuli, except for monkey B, which showed a shortage of palatable food stimuli compared to unpalatable food stimuli (Fig. S5G). The behavior of dropping food had similar challenges as well in the natural food condition (Figs. S5H and S5I). The CA in all internal states “altogether” increased with palatable food stimuli compared to unpalatable food stimuli (Fig. S5J, Movie S4). Hence, for artificial and natural food conditions, animals generally exhibited a higher preference for palatable food than unpalatable food in all internal states.

### 2.5 2D plot visualizations elucidating the effects of hunger and palatability

**Fig. 3:**
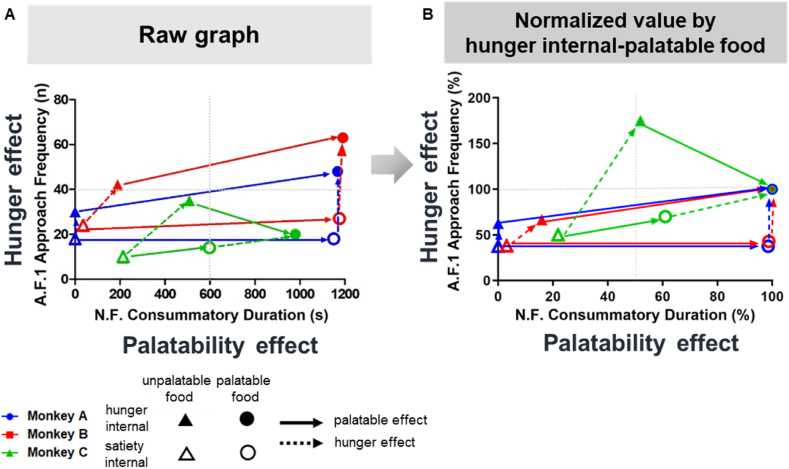
2D plot visualizations elucidating the effects of hunger and palatability. Manual analyses indices. **(A)** Raw data for the 2D plot visualization. The representative index for palatability was M-CD in the natural food condition and for hunger, M-AF in artificial food condition 1 (A F.1; artificial food condition 1, N·F.; natural food condition). **(B)** Normalized 2D plot visualization derived by dividing the index by the hunger internal-palatable food value are shown. This indicates the hunger and palatability effect on predicting the state of the monkeys whether they were hungry and preferred.

To further elucidate the effects of hunger and palatability in Fig. 2, we developed 2D visualization plots based on manual indices (Fig. 3). We selected the M-AF in artificial food condition 1 for the hunger effect and M-CD in the natural food condition for palatability based on the rate of change that most reflected the effect on hunger and palatability, respectively. We then plotted these indices in a 2D plot: palatability-specific index on the x-axis and hunger-specific index on the y-axis (Fig. 3A). Therefore, the plot with M-CD in the natural food condition as a palatability effect (x-axis) and M-AF in the artificial food condition 1 as a hunger effect (y-axis) were shown. Aligned on the x- and y-axes, palatability and hunger values were arranged by internal and food factors; the 2D plot of each monkey had a similar pattern and shape. Furthermore, a normalized 2D plot was generated (normalized value derived by dividing the index by the hunger internal-palatable food value; Fig. 3B).

Collectively, the 2D visualization method we developed could intuitively depict the palatability perception and hunger internal state of the four results (hunger internal-palatable food, hunger internal-unpalatable food, satiety internal-palatable food, and satiety internal-unpalatable food).

### 2.6 The consistency between manual and deep learning-based analyses of between M-AD and D-AD, and between M-AF and D-AF

**Fig. 4:**
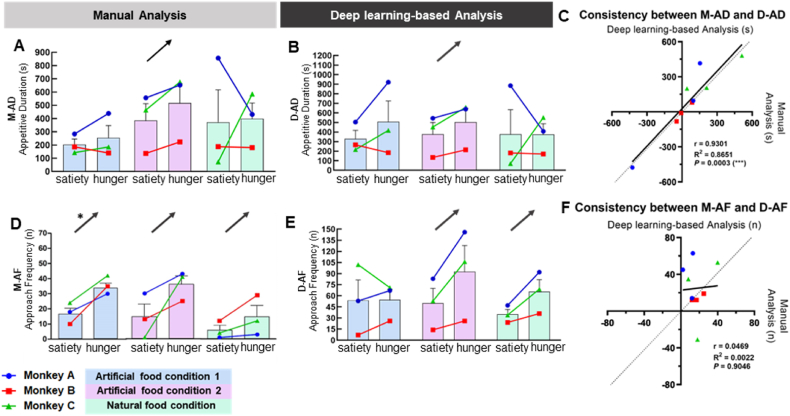
The consistency between manual and deep learning-based analysis between M-AD and D-AD, and between M-AF and D-AF. The consistency between **(A)** Manual analysis index of M-AD and **(B)** Deep learning-based analysis index of D-AD. **(C)** The consistency between M-AD and D-AD. The difference between hunger and satiety internal state (hunger effect) was plotted that all the experimental conditions had a strong positive correlation (r = 0.9301, R^2^ = 0.8651, *P* = 0.0003 (***)), suggesting of robust tendency. The consistency between **(D)** Manual analysis index of M-AF (artificial food condition1, *P* = 0.0395 (*)) and **(E)** Deep learning-based analysis index of D-AF. Similar to appetitive duration, approach frequency index was consistence between manual and deep learning-based analysis in hunger effect. **(F)** The consistency between M-AF and D-AF (r = 0.0469, R^2^ = 0.0022, *P* = 0.9046). The black arrows on the graph indicate that all the monkeys have a same direction (upward or downward) partially along with the *p-value*; *P* < 0.05 (*) and *P* < 0.01 (**). The absolute *P* value is shown on Table S2.

To investigate the consistency between manual and deep learning-based analysis, we compared the behavior indices between M-AD and D-AD, and between M-AF and D-AF. Hunger effect using unpalatable food stimuli was used for the comparison. The hunger effect of deep learning-based analysis using unpalatable food, all three monkeys exhibited consistent change in the same direction on some food conditions; however, these observations did not obtain statistical significance (Fig. 4B and E). As similar behavior was encoded for the appetitive duration indices, M-AD and D-AD were compared (Fig. 4A–C). In addition, approach frequency of M-AF and D-AF were compared (Fig. 4D–F). The hunger effect (difference between hunger internal state and satiety internal state) on appetitive duration index was consistent (same direction of change for all 9 trials) between manual and deep learning-based analysis with significant strong positive correlation (M-AD and D-AD; r = 0.9301, R^2^ = 0.8651, *P* = 0.0003 (***)) (Fig. 4C). Regarding approach frequency (M-AF and D-AF), 8 out of 9 trials showed the same direction of change for hunger effect. However, the correlation between M-AF and D-AF was not statistically significant (Fig. 4D–F).

To investigate if there is a significant difference between manual and deep learning-based analyses, we conducted an analysis using Bland-Altman difference plot (Fig. S6). The absolute differences between M-AF and D-AF (Fig. S6A, 95 % Limit of agreement: −339.0–339.3), M-AD and D-AD (Fig. S6B, 95 % Limit of agreement: −328.2–18.28) demonstrated that most of the variance of difference was limited to an acceptable range of 95 % confidence intervals. In addition, the difference of hunger effect (calculated by subtracting satiety internal state from hunger internal state) between M-AF and D-AF (Fig. S6C, 95 % Limit of agreement: −545.8–479.7), M-AD and D-AD (Fig. S6D, 95 % Limit of agreement: −360.8–149.8) were also remained within an acceptable range of 95 % confidence intervals. In summary, most of the absolute and hunger effect difference values lay within an acceptable limits range.

When comparing the time required for analysis, deep learning-based analysis was faster than manual analysis. In contrast to the manual analysis based on human observation, deep learning-based analysis automatically analyzed the body part coordinates throughout the whole video, based on the pre-defined operational definitions. For example, manual analysis typically required 720 min per video set (12 videos of 20 min duration, 60 min/video). In contrast, the deep learning-based analysis took only 30 min (including labeling time) for the same set of videos (2.5 min/video), which is approximately 24-times faster.

Collectively, these results conclude that we developed a deep learning-based analysis that is accurate and comparable to manual analysis with better efficiency.

### 2.7 Temporal dynamics of 5-min bins measured by manual analysis

**Fig. 5:**
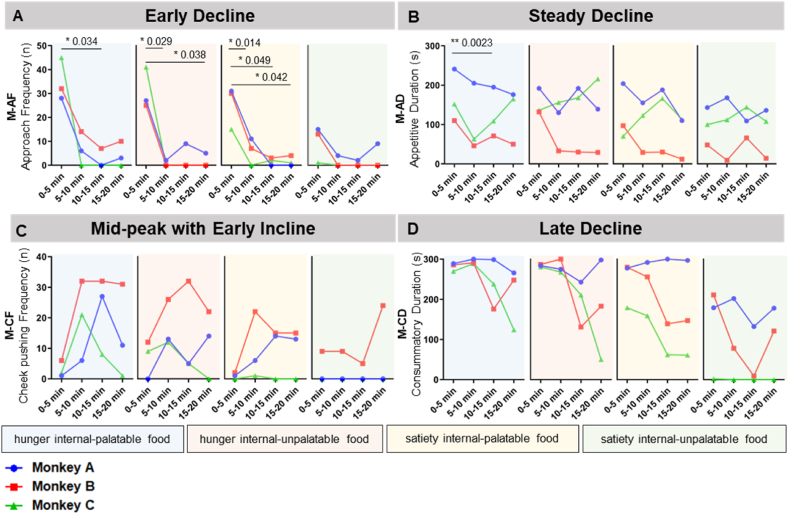
Temporal dynamics of 5-min bins measured by manual analysis. A total of 20 min experimental time was segregated every 5 min by explicating the manual analysis. The temporal dynamics analyses were conducted with artificial food condition 2. A representative index of suitable food for each category was arranged. **(A)** The representative index for classification of “early decline” was M-AF. **(B)** The representative index for classification of “steady decline” was M-AD. **(C)** The representative index for classification of “mid-peak with early incline” was M-CF. **(D)** The representative index for classification of “late decline” was M-CD. *p-value*; *P* < 0.05 (*) and *P* < 0.01 (**).

To investigate temporal dynamics of these behaviors, we segregated every 5-min bin based on a total 20 min duration (Fig. 5). Based on temporal dynamics analysis, the patterns were classified into four categories: early decline, steady decline, mid-peak with early incline, and late decline. The temporal dynamics of behavior indices were classified as early decline when the indices were highest in the first 5 min and then exhibited a rapid decline, signifying that the first 5 min were critical for the eating behavior. The representative index for “early decline” was M-AF (Fig. 5A). As expected, the first 5 min exhibited the most pronounced decline, and this was statistically confirmed as a decrease in results. Furthermore, the temporal dynamics of the behavior index were classified as steady decline when the index exhibited a slow decrease after the first 5 min, contrary to a sharp decrease in early decline. A representative index for steady decline was M-AD in the one-food test, indicating a gradual drop from the first to the last 5 min in all internal state food conditions (Fig. 5B). Similarly, M-AD also demonstrated a statistically significant decrease in the hunger internal-palatable food state. Moreover, the temporal dynamics of the behavior index were classified as mid-peak with early decline when the index initially exhibited an upward trend and peaked in the middle. A representative index for mid-peak with early decline was M − CF, indicating that the monkeys initially pocketed the food inside their cheeks without consumption and then began pushing their cheeks from the middle period (Fig. 5C). Last, the temporal dynamics of the behavior index were classified as late decline when there was no significant change in the early and middle stages, however, a decrease was observed in the last 5 min. A representative index of late decline was M-CD (Fig. 5D). In conclusion, while most values did not exhibit statistically significant results, they could be qualitatively observed through temporal dynamics visualization, resulting in their categorization into four classifications.

### 2.8 Temporal dynamics of eating behavior measured by deep learning-based analysis

**Fig. 6:**
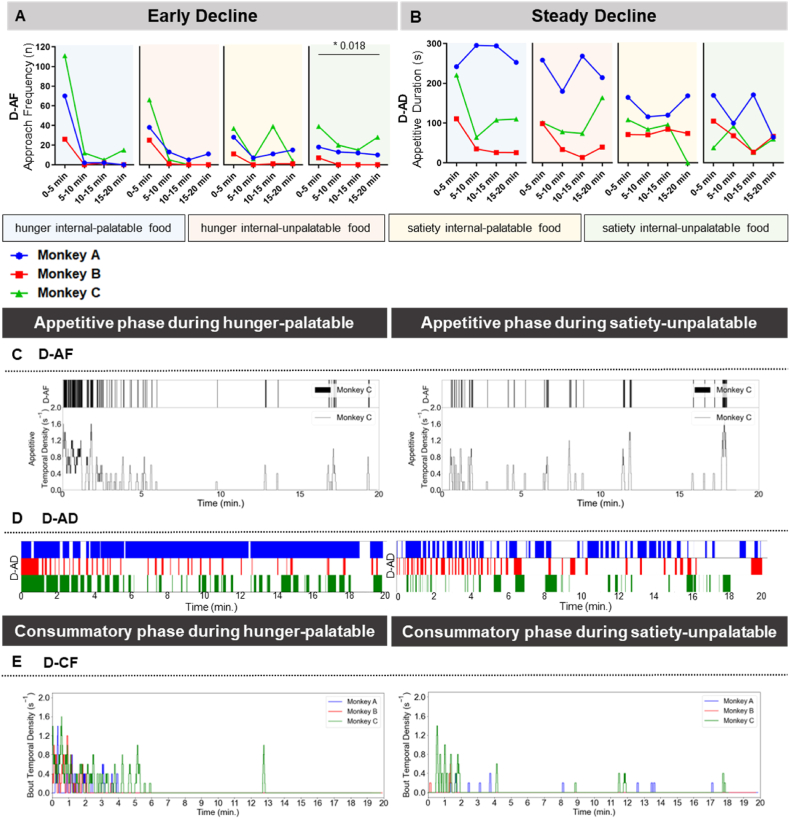
Temporal dynamics of eating behavior measured by deep learning-based analysis. (A) Temporal dynamics of D-indices when divided into 5mins bins. The behavioral index for classification of “early decline” was D-AF. **(B)** Temporal dynamics of D-indices when divided into 5mins bins. The behavioral index for classification of “steady decline” was D-AD. **(C)** The additional temporal distribution of D-AF on Monkey C. Each bar in the upper plot represents moments when a hand approached the tray and the lower plot shows the temporal density of D-AF over time. **(D)** The additional temporal intervals during which each monkey stayed in the food zone, for Monkeys A, B, and C in hunger internal-palatable food (left) and satiety internal-unpalatable food (right). **(E)** The additional temporal distribution of D-CF of Monkeys A, B, and C in hunger internal-palatable food (left) and satiety internal-unpalatable food (right). For each state, the top plot shows the temporal densities of D-CF in each monkey together and the lower ones show the moments of bout and the temporal density of each monkey separately. The temporal dynamics analyses were conducted with artificial food condition 1. *p-value*; P < 0.05 (*).

Similar to manual analysis, the temporal dynamics of events contributing to the deep learning-based indices were also examined. As a result, when we conducted pattern classification analysis, similar to the manual temporal dynamics analysis, we observed that D-AF corresponded to an early decline, while D-AD indicated a steady decline (Fig. 6A and B). In these temporal dynamics, similar to manual, the D-AF exhibits a robust temporal dynamics pattern of “early decline” with the significant statistical result. Furthermore, in our deep learning-based analysis, we employed an additional method for visualizing temporal dynamics. For D-AF, we obtained the temporal density of the tray approach to quantitatively observe their temporal distribution. The temporal density of the tray approach was typically higher in the first 3 min of the experiment than in the later portion (Fig. 6C, Figs. S7B and S7D), which is similar to “early decline” classification of manual analysis (Fig. 5A). Moreover, we plotted the temporal density of the tray approach to quantitatively depict their temporal distribution for the distance between the tray and hand, and the distribution of the tray approach (Figs. S7A and S7C). The “hand in tray” time (defined by below threshold distance between the tray and hands) was higher in hunger internal-palatable food than in satiety internal-unpalatable food; that is, D-AF was higher in hunger internal-palatable food than in satiety internal-unpalatable food (Figs. S7A and S7C: top panel). Next, for D-AD, the temporal intervals at which each monkey remained in the food zone were assessed (Fig. 6D). Similar to D-AF, the earlier intervals were longer than the later ones, indicating that the monkeys focused on the food at earlier times in the experiment. All experimental results are presented in Fig. S8. Finally, for D-CF, the distribution of the bout and its temporal density were visualized (Fig. 6E; temporal densities for all results presented in Fig. S9). Similar to the tray approach, the temporal bout density was generally higher in the first several minutes of the experiments than in the later portion, indicating that the monkeys brought food to their mouths much more frequently in earlier in the study.

In summary, the method developed in this study successfully visualized the appetitive and consummatory phases through deep learning-based analysis by showing temporal dynamics along with the manual analysis. In particular, these deep learning-based outcomes were typically concentrated in the first few minutes and were more frequent in hunger internal-palatable food than in satiety internal-unpalatable food.

### 2.9 Heatmap of spatial dynamics and trajectory-related data measured by deep learning-based analysis

**Fig. 7:**
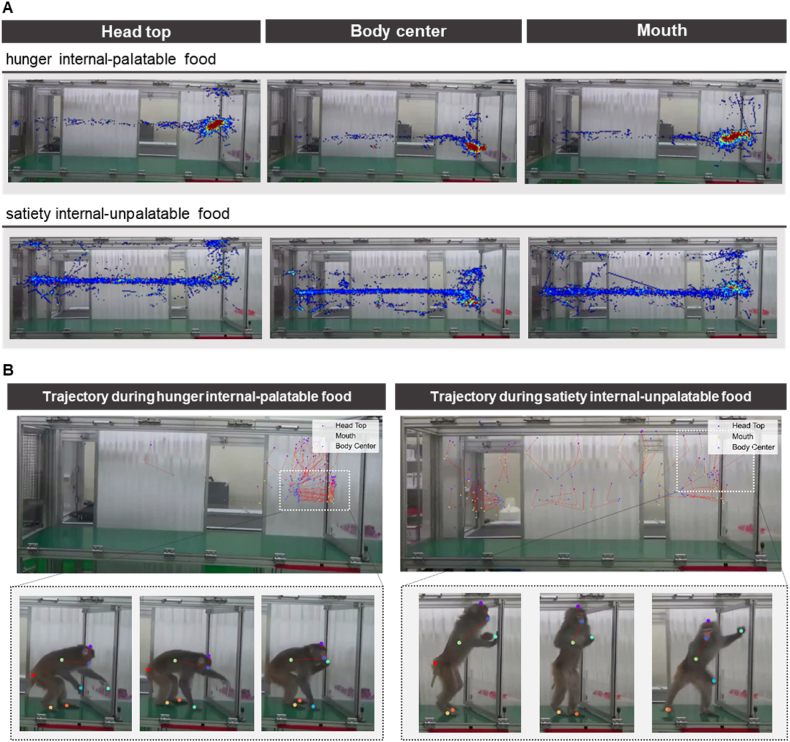
Heatmap of spatial dynamics and trajectory-related data measured by deep learning-based analysis. (A) The proportion of time during which the head top (left), body center (middle), and mouth (right) of Monkey A were labelled at each spatial zone over time, in hunger internal-palatable food (upper) and satiety internal-unpalatable food (lower) states of artificial food condition 1. The red region of the heat-maps refers to a higher proportion and the blue region refers to a lower proportion. **(B)** The spatial positions of the head-top, body center, and mouth of Monkey B in hunger internal-palatable food (left) and satiety internal-unpalatable food (right) states of artificial food condition 1. The plot presents the average positions of the body parts for every 1 s over the first 1 min as dots, with lines connecting head top-mouth and mouth-body center, and representative postures of the monkey on bottom. The red line under the cage of picture indicated the food zone.

To assess spatial dynamics, we visualized the heat map data with a designed experimental cage to determine the accumulated time for which an animal occupied a certain location. We marked the spot of the body part with color density during the total 20 min test. The data were divided into hunger internal-palatable food and satiety internal-unpalatable food for substituting each body part (Fig. 7A). The food zone duration of the body center, head top, and mouth were longer in the hunger internal-palatable food than satiety internal-unpalatable food conditions. Further, the locations of the head top and mouth were closer to the tray than the body center, indicating that the monkey was heading toward the tray within food zone. The results for the other two monkeys are presented in Fig. S10.

Furthermore, we traced the trajectory with dots and lines in the first minute to show the initial tendency (Fig. 7B). The results for the other monkeys are shown in Fig. S11; trajectory video labeling by reflecting the previous 0.3 s is presented in Movie S5 and S6. The red line connected with the average positions of head top-body center-mouth every second for 1 min, enabling the prediction of the posture. Similar to the heat map data, during hunger internal-palatable food, the body position visualization results depicted “consumption posture” pattern (Fig. 7B, left). All body parts were heading toward the tray within food zone as it approached and consumed the food. Contrary to hunger internal-palatable food, when in the satiety internal-unpalatable food condition a standing-up pose was observed with no interest in food in the first minute, without definite “consumption posture” pattern (Fig. 7B, right). Consequently, the heat map and trajectory data showed the location information by spatial dynamics and how the monkeys were more interested in food during the hunger internal-palatable food state than in the satiety internal-unpalatable food state.

## 3 Discussion

We developed freely moving nonhuman primates' eating behavior assessment using manual and deep learning-based analysis. Validation of the palatability and hunger effect also demonstrated the method's usefulness and effectiveness. We found that for hunger effects, M-AF and M-CD were most prominent with unpalatable food, while for palatability effects, M-AC and M-CD were dominant in the satiety internal states. Comparing manual and deep learning methods, deep learning-based analysis is accurate and comparable to manual analysis with better efficiency. In temporal dynamics, we observed that the behavioral indices could be qualitatively classified into four categories. In spatial dynamics, we observed a 'consumption posture' indicating greater interest in palatable food during hunger internal states, with both temporal and spatial aspects emphasizing this preference over satiety internal states.

We developed eating behavior assessment methods for both appetitive and consummatory phases and verified that the M-AF and M-CD indices were most effective for quantification of the hunger effect. Meanwhile, the M-AC score and M-CD indices were most effective for quantifying the palatability effect. We also verified that our deep learning-based analysis method provides excellent quantification data with high temporal resolution and comprehensive spatial and temporal information with minimal labor.

While previous papers have observed the eating behavior within natural environments [[14], [15], [16]], and in the context of social behavior [12,13,42], behavioral change [21], and head-fixed conditions [[46], [47], [48], [49]], we developed experimental assessment methods in moved freely using a manufactured cage and food. As such, our model has certain benefits. First, the developed methods were tested rigorously to validate their utility for measuring the hunger and palatability effects. Second, the methods to measure eating behavior in nonhuman primates were based on deep learning. Additionally, we processed and interpreted the raw output of DeepLabCut (e.g., linear interpolation or minimum-interval-constraint) to alleviate the effect of outliers and facilitate more accurate analyses of the actual behaviors. Third, we developed an eating behavior assessment cage designed to specifically focus on eating behavior under freely moving conditions (not fixed in a chair; Fig. 1). We were also able to obtain more detailed results by incorporating an artificial jelly, for which the flavor and tactile texture were controllable with the taste only altered based on sugar concentration (Fig. S1). Finally, we compared the similar encoded behavior of approach frequency (M-AF and D-AF) and appetitive duration (M-AD and D-AD) between manual and deep learning-based analysis. Indeed, the deep learning-based analysis accurately quantified behavior, which was comparable to manual analysis, however, with faster analysis times (Fig. 4).

In addition, the eating behavior analysis method developed in this study has several research applications. First, for neuroscience and ethology studies, this method can be used to analyze and quantify the palatability value of a specific food using nonhuman primates, thus, allowing for more accurate prediction of human responses. Second, by examining the internal state effect, the effect of hunger can be analyzed in the field of homeostasis. Third, for the application of manual indices, the internal state and palatability cognition of a monkey can be predicted using the developed 2D plot visualization. For instance, if a monkey is plotted in the upper right corner of Fig. 3, it could be interpreted as high palatability–hunger internal; conversely, if a monkey is in the lower-left corner, it could be interpreted as low palatability–satiety internal. Fourth, the eating behavior of temporal dynamics can be predicted by our temporal dynamic data. For example, based on the early decreasing pattern of M-AF, we may assume that analyzing only the first 5 min of tests will be sufficient to quantify M-AF. Finally, the deep learning-based indices can be applied with the following advantages. These indices are based on the predicted coordinates of the body parts, which facilitate the generation of explicit operational definitions for each index. For instance, our definition of D-AF involves the distance between the hand and tray and the minimum interval constraint. In addition, each researcher can assign their own operational definitions and apply them as indices. Especially, the researcher can change all DeepLabCut parameters to enhance the effectiveness. Indeed, the present DeepLabCut parameters are optimized to maximize their effectiveness and accuracy. Furthermore, when compared to manual analysis, the amount of labor and time required to obtain the indices is significantly reduced for deep learning-based analysis. Moreover, the indices for each experiment are paralleled between the two analysis methods so that a comparison between experiments can be provided with high confidence.

A biologically intriguing result was observed when comparing the effects of hunger using unpalatable food or palatable food (Fig. 2, and Fig. S3). That is, unpalatable food stimuli demonstrated a better hunger effect than palatable food stimuli, considering that unpalatable food obtained a higher total score than palatable food (Fig. 2A and C, Table S1). As expected, palatable food driven hedonic eating was sufficiently consumed independent of hunger level. In contrast, unpalatable food driven homeostatic eating required a high level of hunger to seek and consume; that is, dependent on hunger level [50]. Similarly, the palatability effect was more evident in the satiety internal state than in the hunger internal state, considering that satiety internal state food obtained a higher total score than hunger internal state (Fig. 2B and D, Table S1).

Including the hunger and palatability, the sensory processes such as the sense of smell may also be considered as a key factor in appetite, food choice and intake, which is to consider the metabolic processes as one of the key factors in eating behavior. Based on the several studies [[51], [52], [53]], odors’ primary role during the anticipation phase of eating behavior are to identify food sources in the living environment and induce appetite. The significance or preference of the smells, however, can vary depending on culture and label indicating that they may be adaptable and associative [51]. We used food stimuli that are similar in exposure to nonhuman primates but have the least odor to control the variability in the learned associations. Therefore, this study is focused on investigating the eating behavior driven by the hunger (metabolic processes) and palatability by validating the efficacy and utility of tools.

Certain limitations were noted in this study. First, the number of animals was small (*n* = 3). Due to the limited resources of the nonhuman primate, we were unable to extend the sample size. Several previous studies reported important scientific findings using one or two nonhuman primates [54,55]. In addition, to overcome this sample size limitation, we selected primates with relatively similar tempers based on the temperament test [56]. Second, the limited number of subjects included in our study has weakened the significant statistical result of our findings. Furthermore, given the multitude of statistical tests conducted in our research, utilizing a significance cutoff of P < 0.05 without applying adjustments for multiple comparisons increases the susceptibility to false positive results.

Overall, the development of a nonhuman primate eating behavior assessment method using both manual and deep learning-based analyses provides novel applications for the analysis of nonhuman primate ethology and research related to reward, homeostasis, eating behavior, and obesity. In addition, we successfully verified its performance by applying it to hunger and palatability effects.

## 4 Methods

### 4.1 Experimental animals

Three young (aged 5–6 years) female rhesus monkeys (monkeys A, B, and C), based on the age classification standards for humans and macaques [57,58], were included in this study. The three animals had similar tempers, based on the results of a temperament test described previously [56]. All procedures were approved, and conducted according to the guidelines set, by the [BLINDED FOR REVIEW] Institutional Animal Care and Use Committee (Approval No. [XXXX]) and conformed to the Animal Research Reporting of In Vivo Experiments (ARRIVE) guidelines [59] and National Institutes of Health guidelines in the USA. The monkeys were obtained from [BLINDED FOR REVIEW] and were maintained in indoor cages at the [BLINDED FOR REVIEW]. They were fed commercial monkey chow (Teklad 2050™, Envigo, USA) supplemented with various fruits and were given water *ad libitum*. Environmental conditions were maintained at a temperature of 24 ± 2 °C, relative humidity of 50 ± 5 %, and a 12 h light/12 h dark cycle. The attending veterinarian monitored the monkeys' health in accordance with Weatherall et al.’s report on the use of nonhuman primates in research [60]. Microbiological tests for B virus, simian retrovirus, simian immunodeficiency virus, simian virus 40, and simian T-cell lymphotropic virus were performed once per year, as previous paper described [61].

### 4.2 General training procedures

The experimental design (CATIA V5, Dassault Systèmes, France) of the chamber is presented in Fig. 1A. Briefly, the testing apparatus comprised a custom-built transparent plexiglass tunnel (195 × 51 × 84 cm) with one or two eating trays (hereafter, designed experimental cage). A single primate transfer cage was docked on the tunnel entrance using a sliding door (Fig. 1A). All monkeys became familiarized with the experimental cage and food stimuli (palatable food and unpalatable food) through a conditioning experiment conducted for 50 days.

### 4.3 Eating behavior test with controlled food access

During the pairwise-food test, each participant was assessed three days per week for 15 min each, while the one-food test was conducted for 20 min. To analyze the hunger internal, in the case of the pairwise-food test, access to food was restricted for 24 h and then for 72 h during the one-food test. Before setting the hunger internal state for the one-food test, we optimized the fasting time by observing when the eggplants were eaten after 24, 48, and 72 h of fasting. It was confirmed that the monkeys ate eggplant (unpalatable food) even after 72 h fasting.

To monitor the animals’ health, a veterinarian monitored food intake and stress or fear behavior throughout the study. Additionally, the monkeys were weighed each day and blood analysis was performed semi-annually. Moreover, regarding the food restriction protocols, food was not restricted until after home cage adaptation was achieved, water was always available, and vitamins and supplements were provided as needed as per the discretion of the veterinarian. Additionally, implementation of fasting was gradual, allowing time for the animals to become aware that their intake is being restricted [62]. Importantly, when body weight decreased by 15–20 % fasting was temporarily halted [63]. Meanwhile, when assessing the satiety internal, additional food was provided after 30 min of eating; during the pairwise-food test to assess neutral internal state, food was supplied at the originally offered time. All experiments were performed between 12:00 and 14:00 (Fig. 1C).

### 4.4 Food stimuli

We developed a manufactured jelly that was differentiated by sugar concentration (levels 1 to 4). In the conditioning phase, the jellies were tested on monkeys to distinguish between unpalatable food and palatable food stimuli (hereafter, artificial food; Fig. S1). We exposed the monkeys to the artificial food for 1 h in the home cage, which they perceived as a comfortable environment. As expected, the monkeys perceived level 1 jelly (tasteless) as unpalatable food stimuli and level 4 jelly (palatable) as palatable food stimuli. However, since the monkeys did not consume any level 1 jellies (which contain 0 g sugar), level 2 jelly was applied as the unpalatable food stimuli. The experiment was performed twice and designated artificial food condition 1 and artificial food condition 2. In addition to artificial food, natural food, such as fruits, were used. We first used cucumber, eggplant, and cherry and conducted the same pre-test as that for artificial food to select the unpalatable food and palatable food stimuli. Consequently, eggplant and cherry were selected as the unpalatable food and palatable food stimuli, respectively. In summary, we used level 2 jelly in the artificial food conditions and eggplant in the natural food condition as unpalatable food stimuli, while level 4 jelly in the artificial food conditions and cherry in the natural food condition served as the palatable food stimuli. That is, the total number of trials was 3, with 1 trial conducted using natural food and 2 trials using artificial food. Furthermore, to quantify whether the quantity taken at once varies based on the level of hunger, we divided approach frequency by the total amount of food intake (Fig. S12). We found no significant differences in this ratio across different internal states, indicating that the quantity taken by the monkeys at once did not vary based on internal state.

### 4.5 Conditioning experiments for the food and designed experimental cage before the main experiments

To investigate the conditioning level of novel food stimuli, we measured the frequency and latency for choosing each type of food: natural palatable food and unpalatable food stimuli and artificial palatable food and unpalatable food stimuli. In all three monkeys, the frequency and latency of palatable food and unpalatable food stimuli for artificial food conditions increased in the last trial compared with the first trial. Additionally, the frequency of natural palatable food consumption increased in the last trial compared with the first trial. Hence, all monkeys were conditioned to the food stimuli, implying that they could interact with novel food stimuli for the eating behavior test. The conditioning experiment results are presented in Fig. S2. No significant difference in motor activity was observed between the home cage and experimental cage.

### 4.6 Pairwise-food test for verification of food preference applied on manual eating behavior indices

Similar to the one-food test, eating behavior analysis for the pairwise-food test was classified into two phases. For the appetitive phase, four indices were developed: frequency of food approach (M-AF, approach frequency), latency to first approach (M-AL, approach latency), number of food drops (M-AF-d, approach frequency-drop), and latency to first drop (M-AL-d, approach latency-drop). For the consummatory phase, one index was developed: the amount of food intake (CA, consummatory amount).

A pairwise-food test was conducted by placing both palatable food and unpalatable food stimuli on the tray to assess the preference and eating behavior as stated above. Both artificial and natural food conditions were used for the experiment (Fig. 1B right panel). Both artificial and natural food conditions were analyzed by behavior indices, and each index was analyzed by generating appetitive (Figs. S5A–D, S5F–I) and consummatory phases (Figs. S5E and S5J). In M-AL and M-AL-d, a wave indication >900 s implied that the monkey had never touched the food.

### 4.7 Analysis method

#### 4.7.1 Manual analysis

During the experiment, the behaviors of each monkey were simultaneously recorded by side and front cameras (HDR-CX405, SONY, Japan). All cameras were synchronized by handclaps at the start of the experiment (camera sampling rate: 30 frame/s). With the recorded video, the Noldus Observer XT program was used for analysis, and all behaviors were observed at 0.5 × –1 × speed (Movies S1–S4; 1 × speed). We divided the coding scheme into two phases—appetitive and consummatory—and both one- and pairwise-food test behavior indices were applied by time event and time duration (Fig. 1D). A total of four manual analysts conducted the analysis according to the behavior indices template. For accurate and unbiased analysis, firstly, all four analysts underwent training on the analysis methodology. Secondly, two manual analysts were paired for each experimental task and performed a double-check analysis, which the two analysts independently cross-validated each other's analyses to ensure the accuracy of the recorded behaviors, while confirming the similarity between each analysis. Fig. 1D–Movie S1 (hunger internal-palatable food), and Movie S2 (satiety internal-unpalatable food) show the observation time between 115 s and 160 s for the artificial food condition 2 with Monkey B (black box).

#### 4.7.2 Deep learning-based analysis

For the deep learning-based analysis method, we used DeepLabCut, which is a markerless pose estimation tool based on a deep neural network. To label and predict the coordinates of each body part with DeepLabCut, we manually extracted 240 characteristic frames for labeling. The body parts labelled with head-top, mouth, body center, both hands, both feet, and tail. The extracted images were transformed into training sets on which the deep neural network was trained. The network comprised ResNet-50 (pre-trained on ImageNet) and deconvolutional layers, the outputs for which are score maps representing the soft predictions for the location of each body part. The network was trained through 500,000 iterations, minimizing the cross-entropy of the predicted probability distribution relative to the ground-truth probability distribution. After training the model, the body part coordinates were predicted (Fig. 1E–Movie S5 and S6). According to a previous paper [40], 240 frames and 500,000 training sessions were sufficient for predicting the body part coordinates. Furthermore, when we plotted the values of loss against the number of training iterations for our data, we observed that the loss values converged to values close to 0, indicating saturation (Fig. S13).

### 4.8 Computation of deep learning-based indices

Before computing the deep learning-based indices, the predicted labels for the body parts of interest were processed based on their likelihood (i.e., confidence of prediction). To reduce the effect of low-likelihood values, all values with a likelihood <0.7 (which means 70 % accuracy) were removed and replaced with new values obtained by linear interpolation using the remaining values—that is, the values with a likelihood ≥0.7 (which means 70 % accuracy). Data with accuracy lower than 70 % were removed as a missing data. After removing the missing data, linear interpolation was performed to fill in the missing data. The video S5 and S6 included the data based on a 70 % accuracy threshold. The video quality was improved by adding estimated points using linear interpolation to fill in the missing points. To obtain deep learning-based approach frequency (D-AF), we first observed the moments in which each hand of the monkey entered the tray zone, which is a circle centered at the tray. When determining the radius of the tray zone circle, 1 cm corresponded to approximately 5.77 px. The minimum interval (0.5 s) of temporally adjacent events for the same hand entering the circle was set. Hence, if DeepLabCut predicted that the same hand entered the zone multiple times in a short time interval (0.5 s), it was unlikely that this occurred. Rather, this was likely caused by incorrect predictions for the specific frames; thus, only the first of the multiple events was counted. After applying this constraint, D-AF was defined as the number of frames corresponding to events of the right or left hand entering the tray zone throughout the video. By introducing this minimum-interval constraint, we prevented overcounting, making the analysis more compatible with reality.

We also computed the temporal density of D-AF. The temporal density of the events at a specific moment is defined as the number of events within the 5 s interval starting from that moment divided by the length of the interval. Next, deep learning-based appetitive duration (D-AD) was defined as the length of time during which the x-coordinate of the body center was larger than that of the left edge of the rectangular food zone. Finally, for deep learning-based consummatory frequency (D-CF), we first observed when the right hand of a monkey entered the mouth zone, i.e., a circle centered at the mouth. The minimum-interval constraint of the interval length 0.5 s was also applied here. For each event of the right hand entering the second circle, we examined whether the right hand was inside the tray zone (the same as used for D-AF) at least once during the previous 3 s. If this was the case, the monkey had extended its right hand to the tray and subsequently brought it to its mouth, strongly implying that this was an action of the right hand. Analogously, we observed when movements by the left hand occurred. Finally, we defined D-CF based on the number of frames corresponding to bouts by the right or left hand. As in D-AF, we also computed the temporal density of D-CF. All the DeepLabCut parameters underwent several processes of optimization to maximize their effectiveness and accuracy.

Since the deep learning-based analysis is based on event time data of body part position, only the frequency of tray approach (D-AF) and the duration in food zone (D-AD) were feasible (latency to first approach to food was not feasible). Due to the limitation of the current spatial resolution of the videos, it was not feasible to distinguish the consummatory behaviors of duration of food chewing (M-CD). Therefore, we could not apply the deep learning-based analysis to M-CD. For D-CF, direct comparison with M − CF was not feasible because of differences in nature (D-CF was bout frequency and M − CF was cheek pushing frequency).

### 4.9 The representative eating behavior indices score

Mathematical calculations were applied to compare and suggest which of the developed indices provided the most efficient and robust performance to quantify the effects of hunger and palatability. A score of one point was assigned when all three monkeys showed same direction (upward or downward directions; black arrow). Statistical significance was also determined, and one point was assigned if the behavior corresponded to a *P* < 0.05, while two points were given if *P* < 0.01 (Table S1).

### 4.10 Statistical analysis

All statistical analyses were performed using SPSS v.26. To compare and analyze whether the two datasets were statistically significant (Fig. 2, Fig. 4, Fig. 5, Fig. 6, Fig. S3–S5, S12), pa ired *t*-tests for two samples were conducted. For the linear regression (Fig. 4) and Bland-Altman plot (Fig. S6), we used GraphPad v.9.3.1 for the analysis. As a result of correlation coefficients, we obtained the value of Person r, R squared, and P value (two-tailed) for the linear regression and Bias, SD of bias, and 95 % Limit of agreement for the Bland-Altman plot analysis. *P*-values <0.05 were considered significant. Identical results for all three monkeys showing same direction were indicated with a black arrow on the graph and “altogether” in the Results section. Absolute *P*-values are provided in Table S2.

For the manual analysis graphs, GraphPad v.9.3.1 was used to display the data, and each value represented mean + standard error of the mean (SEM).

### 4.11 Data and code availability

Data to reproduce the figures is available as of the date of publication at https://github.com/Gavroche11/FeedingIndex for the code related to Fig. 6, Fig. 7, and Figs. S7–S11. All other data are available in the main text or supplemental information. Any additional information required to reanalyze the data reported in this paper will be available from the lead contact upon request.

## Data and materials availability

See https://github.com/Gavroche11/FeedingIndex for the code using Python related to defining deep learning-based indices, computing temporal densities, and producing Fig. 6, Fig. 7, Figs. S7–S11 and Movies S5-S6.

## Ethics approval

All procedures were approved, and conducted according to the guidelines set, by the Korea Research Institute of Bioscience and Biotechnology Institutional Animal Care and Use Committee (Approval No. KRIBB-AEC-20080) and conformed to the Animal Research Reporting of In Vivo Experiments (ARRIVE) guidelines. The monkeys were obtained from Guangzhou Monkey King Biotechnology Co. Ltd. (Guangdong Province, China) and were maintained in indoor cages at the National Primate Research Center at the Korea Research Institute of Bioscience and Biotechnology (KRIBB)

## CRediT authorship contribution statement

**Leslie Jaesun Ha:** Writing – review & editing, Writing – original draft, Visualization, Validation, Project administration, Methodology, Investigation, Formal analysis, Data curation, Conceptualization. **Meelim Kim:** Writing – review & editing, Writing – original draft, Methodology, Investigation, Formal analysis, Conceptualization. **Hyeon-Gu Yeo:** Writing – original draft, Validation, Methodology, Investigation, Formal analysis, Data curation, Conceptualization. **Inhyeok Baek:** Writing – original draft, Visualization, Validation, Software, Methodology, Formal analysis, Data curation. **Keonwoo Kim:** Investigation, Formal analysis. **Miwoo Lee:** Methodology, Formal analysis. **Youngjeon Lee:** Writing – original draft, Supervision, Resources, Project administration, Conceptualization. **Hyung Jin Choi:** Writing – review & editing, Writing – original draft, Supervision, Project administration, Funding acquisition, Conceptualization.

## Declaration of competing interest

The authors declare that they have no known competing financial interests or personal relationships that could have appeared to influence the work reported in this paper.
