# Supplementary material for: Development of an assessment method for freely moving nonhuman primates’ eating behavior using manual and deep learning analysis
Source: Heliyon. 2024 Feb 5;10(3):e25561. doi: 10.1016/j.heliyon.2024.e25561 (PMC10865331; doi:10.1016/j.heliyon.2024.e25561)
Supplement: Multimedia component 1 [file mmc1.docx]

Supplementary Materials for

Development of an Assessment Method for Freely Moving Nonhuman Primates’ Eating Behavior Using Manual and Deep Learning Analysis

**This file includes:**

Supplementary Figs. S1 to S13

Supplementary Table S1 and S2

**Other Supplementary Materials for this manuscript include the following:**

Movies S1 to S6

**
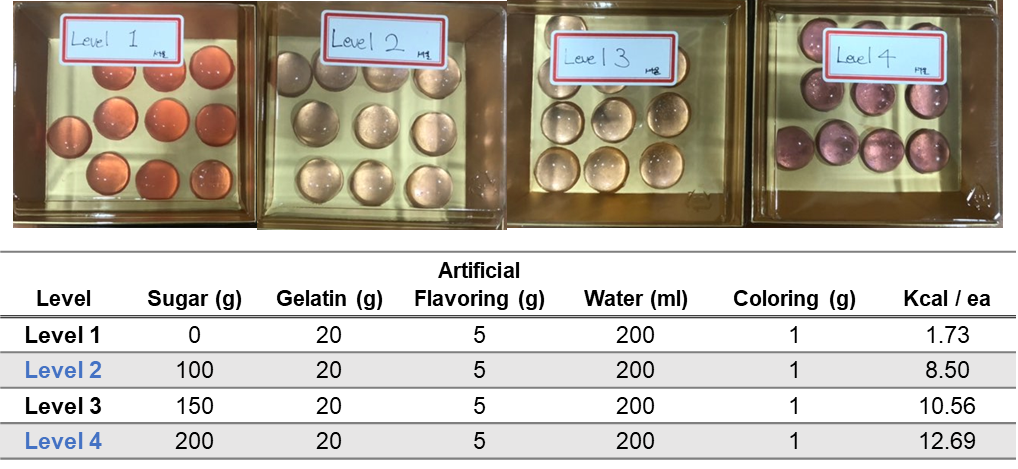
 Fig. S1. Ingredient and picture of artificial food depending on the level.** The manufactured jelly served as an artificial food that was differentiated by its sugar concentration, as shown in the table. While conditioning experiments were executed, Level 2 jelly was selected as an unpalatable food and Level 4 jelly was chosen as a palatable food. All the jellies had the same grape flavor and chewing texture, but only the color and sugar concentration were set differently.

**
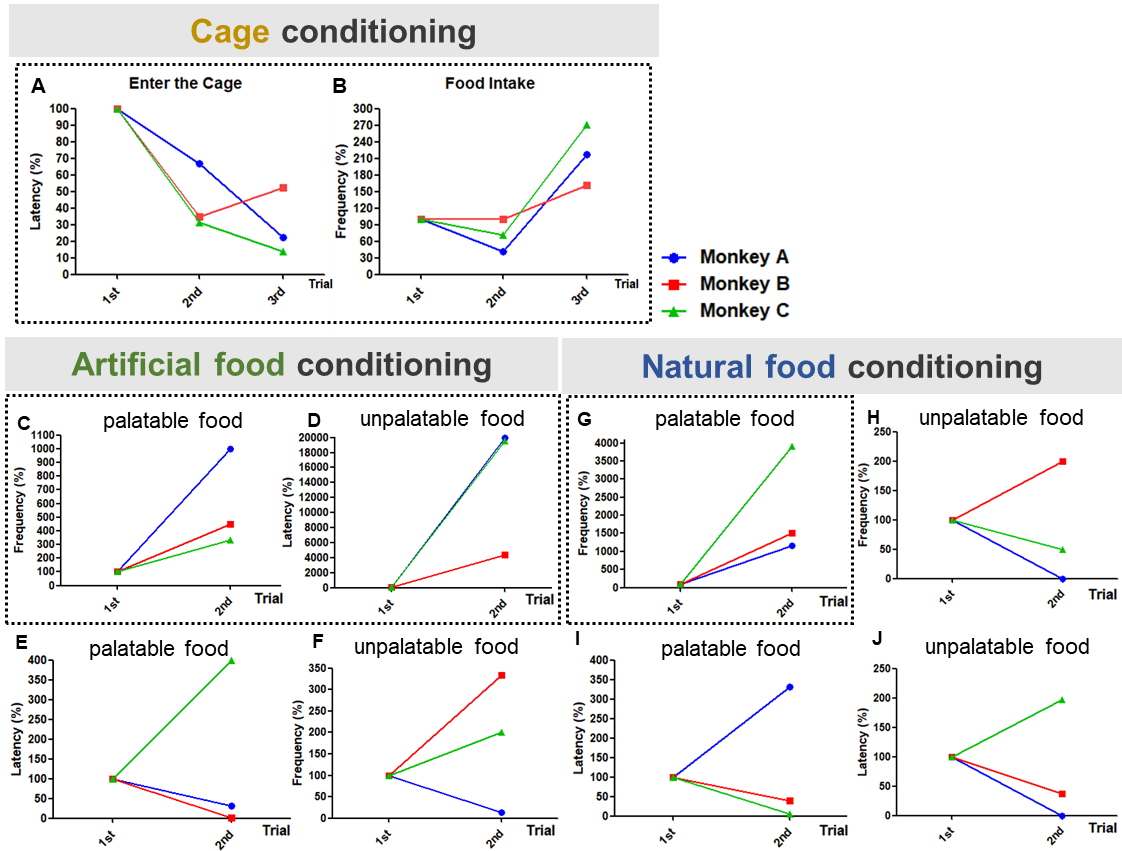
Fig. S2. Conditioning experiments for the food and the designed experimental cage before main experiments.** (**A-B)** Cage conditioning for main experiments. To prove that the monkeys had adapted to the designed experimental cage, several indices (e.g., entering the cage, food intake) were analysed. As the trials were repeated, the indices showed that the monkeys were well-adapted (similar tendency presented on the black dotted box); latency of entering the cage had decreased, and frequency of food intake had increased. **(C-F)** Artificial food conditioning for main experiments. Both palatable and unpalatable foods were well adapted according to their frequency and latency (common tendency presented on the black dotted box); the frequency of the palatable food approach had increased, nonetheless, the latency of the unpalatable food approach had decreased, implying that the monkeys were “trying not to touch” as the trials passed conjecturally owing to their vapidity. (**G-J)** Natural food conditioning for main experiments. Palatable food was well adapted which was evident from the frequency of consumption (common tendency presented on the black dotted box). Similar to artificial food conditioning, no common tendency of indices was found with unpalatable food stimuli.

**
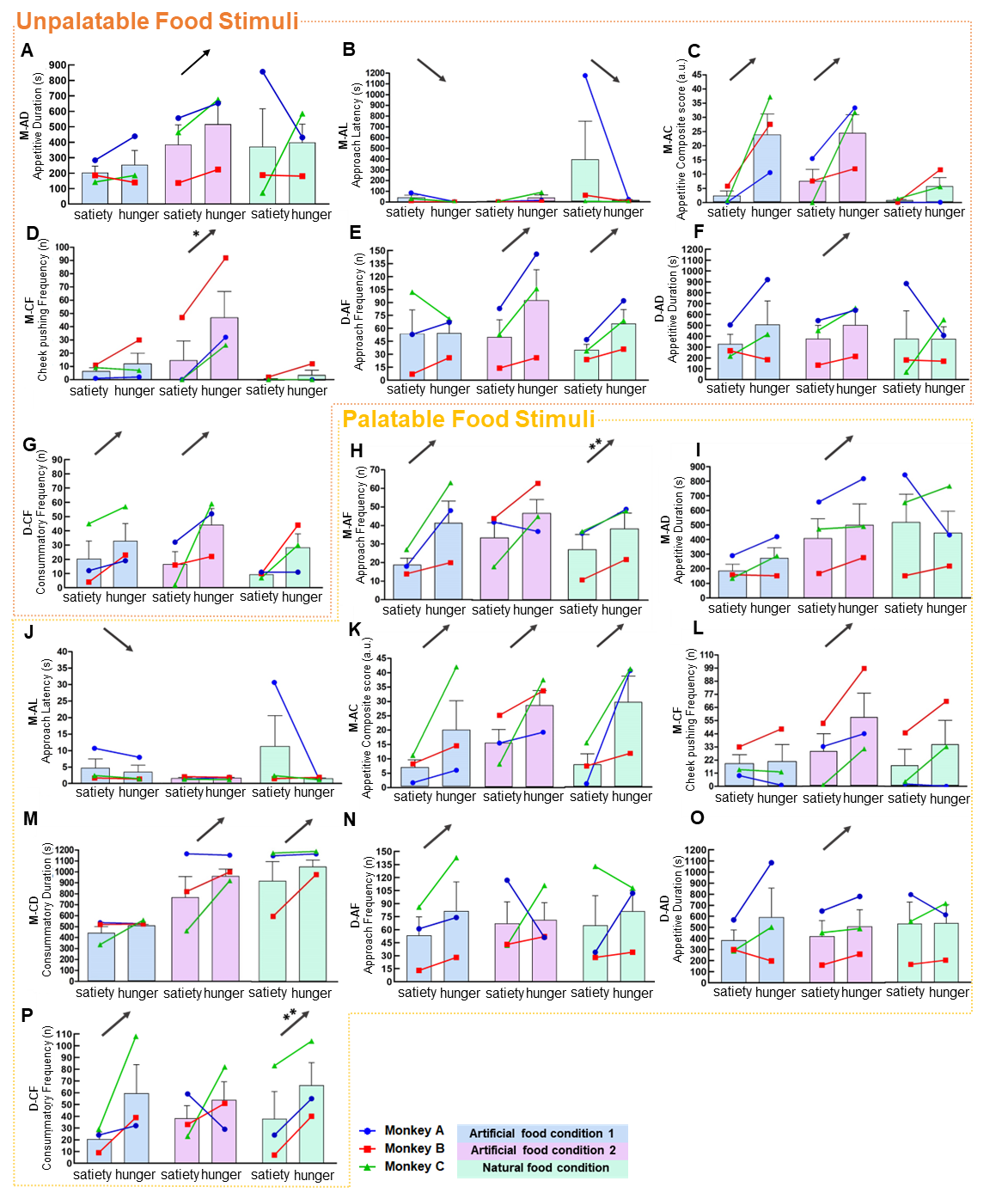
Fig. S3. The supplementary data of hunger effect. (A-C)** Appetitive phase and **(D)** Consummatory phase measured by manual analysis using unpalatable food stimuli. **(E-F)** Appetitive phase and **(G)** Consummatory phase measured by deep learning-based analysis using unpalatable food stimuli. **(H-K)** Appetitive phase and **(L-M)** Consummatory phase measured by manual analysis using palatable food stimuli. **(N-O)** Appetitive phase and **(P)** Consummatory phase measured by deep learning-based analysis using palatable food stimuli. The black arrows on the graph indicate that all the monkeys have a common direction (upward or downward) partially along with the p-value; p<0.05 (*) and p<0.01 (**).

**
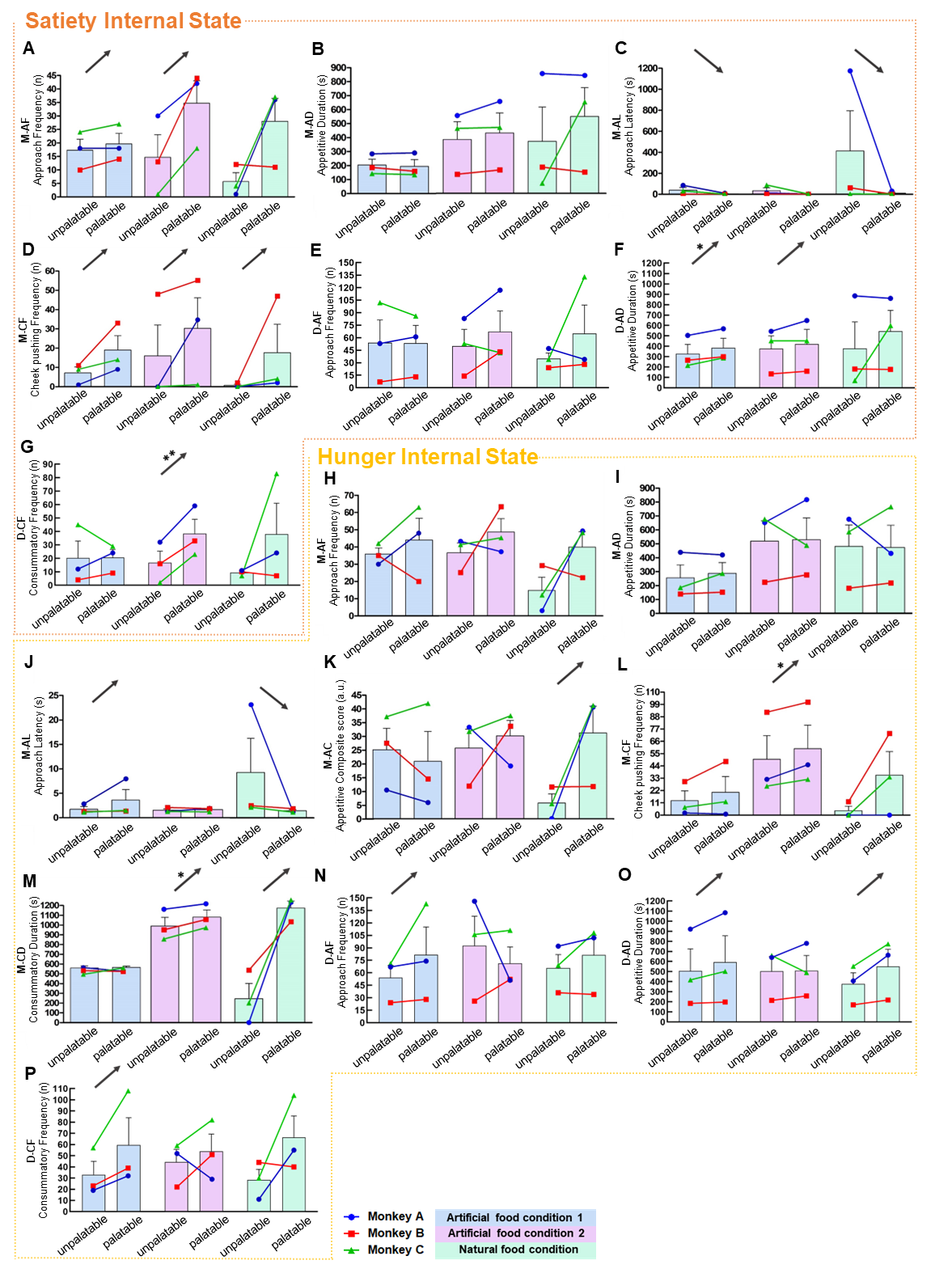
**

**Fig. S4. The supplementary data of palatability effect. (A-C)** Appetitive phase and **(D)** Consummatory phase measured by manual analysis on satiety internal state. **(E-F)** Appetitive phase and **(G)** Consummatory phase measured by deep learning-based analysis on satiety internal state. **(H-K)** Appetitive phase and **(L-M)** Consummatory phase measured by manual analysis on satiety internal state. **(N-O)** Appetitive phase and **(P)** Consummatory phase measured by deep learning-based analysis on satiety internal state. The black arrows on the graph indicate that all the monkeys have a common direction (upward or downward) partially along with the p-value; p<0.05 (*) and p<0.01 (**).

**
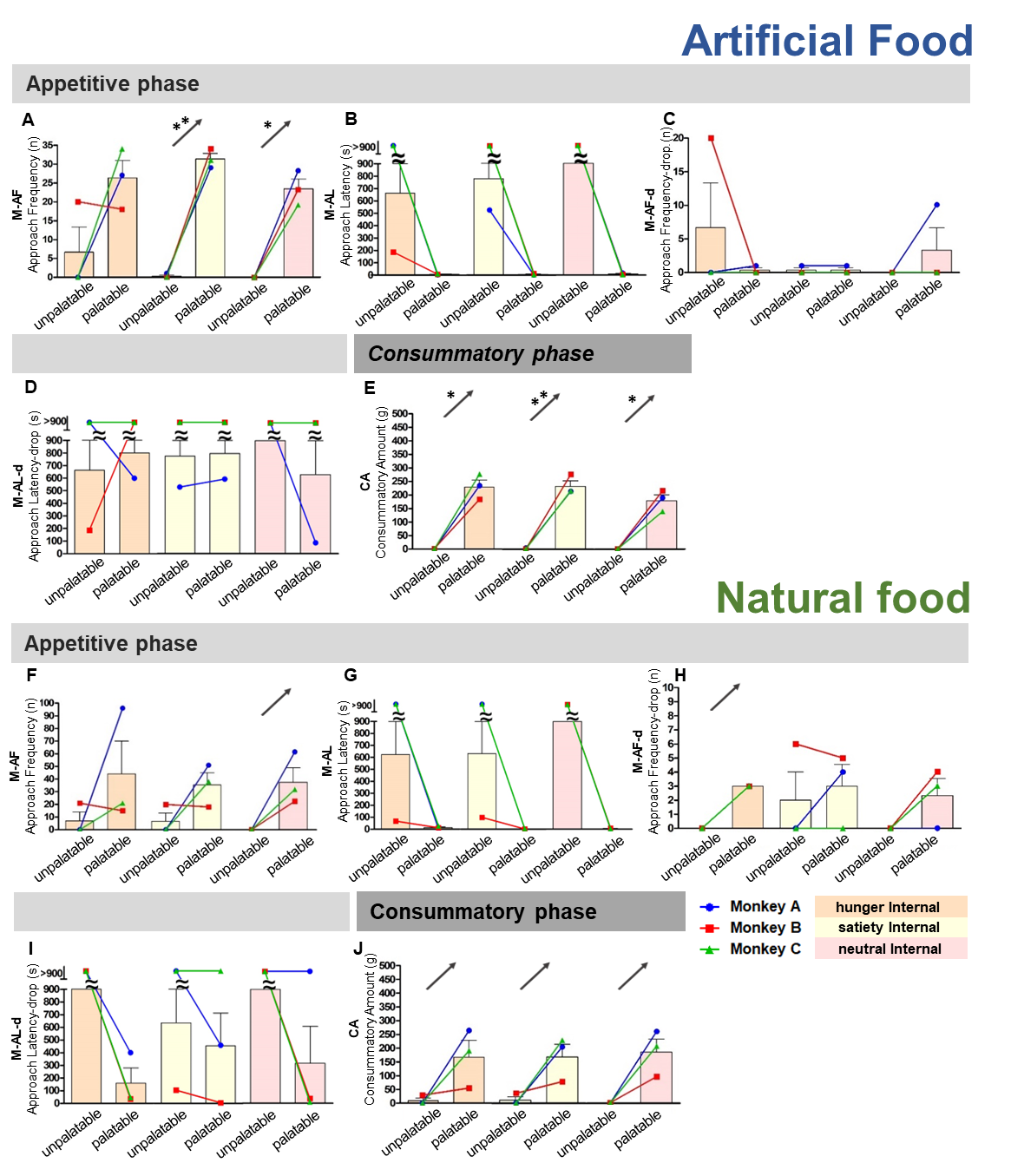
Fig. S5. Pairwise-food test for verification of food preference.** Manual pairwise-food test indices for appetitive **(A–D)** and consummatory **(E)** phases supplied with artificial food stimuli. Together with artificial food, natural food for the manual pairwise-food test indices are represented in **(F–I)** as the appetitive phase, and **(J)** as the consummatory phase. A light-grey tone expressed the appetitive phase, and a grey color tone expressed the consummatory phase. The black arrows in the graph indicate that all the monkeys have a common direction (upward) partially along with the p-value; p<0.05 (*) and p<0.01 (**). Wave indication on the graphs meant more than 900 s, suggesting that they never touched food.

**
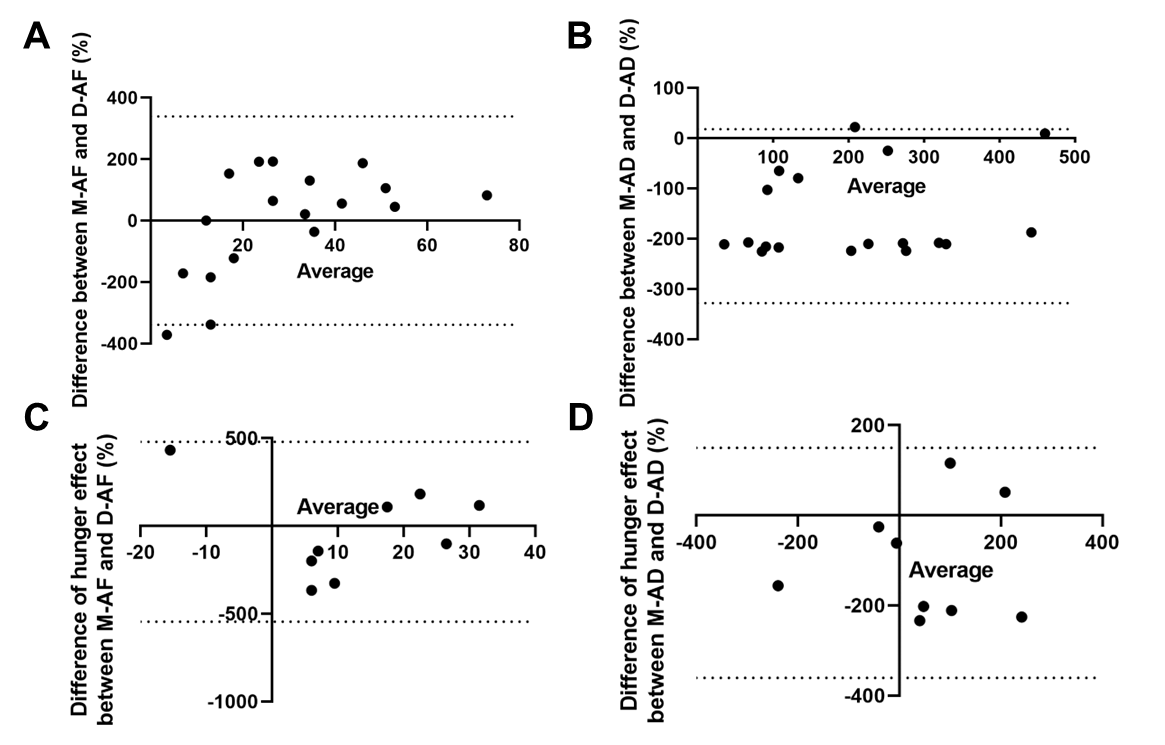
**

**Fig. S6. Bland-Altman plot of difference between M-AF and D-AF, M-AD and D-AD.** **(A)** Absolute difference between M-AF and D-AF (Bias: 0.1847, SD of bias: 173.0, 95% Limit of agreement: -339.0~339.3). **(B)** Absolute difference between M-AD and D-AD (Bias: -155.0, SD of bias: 88.39, 95% Limit of agreement: -328.2~18.28). **(C)** Difference of hunger effect (hunger-satiety) between M-AF and D-AF (Bias: -33.02, SD of bias: 261.6, 95% Limit of agreement: -545.8~479.7). **(D)** Difference of hunger effect (hunger-satiety) between M-AD and D-AD (Bias: -105.5, SD of bias: 130.3, 95% Limit of agreement: -360.8~149.8).

**
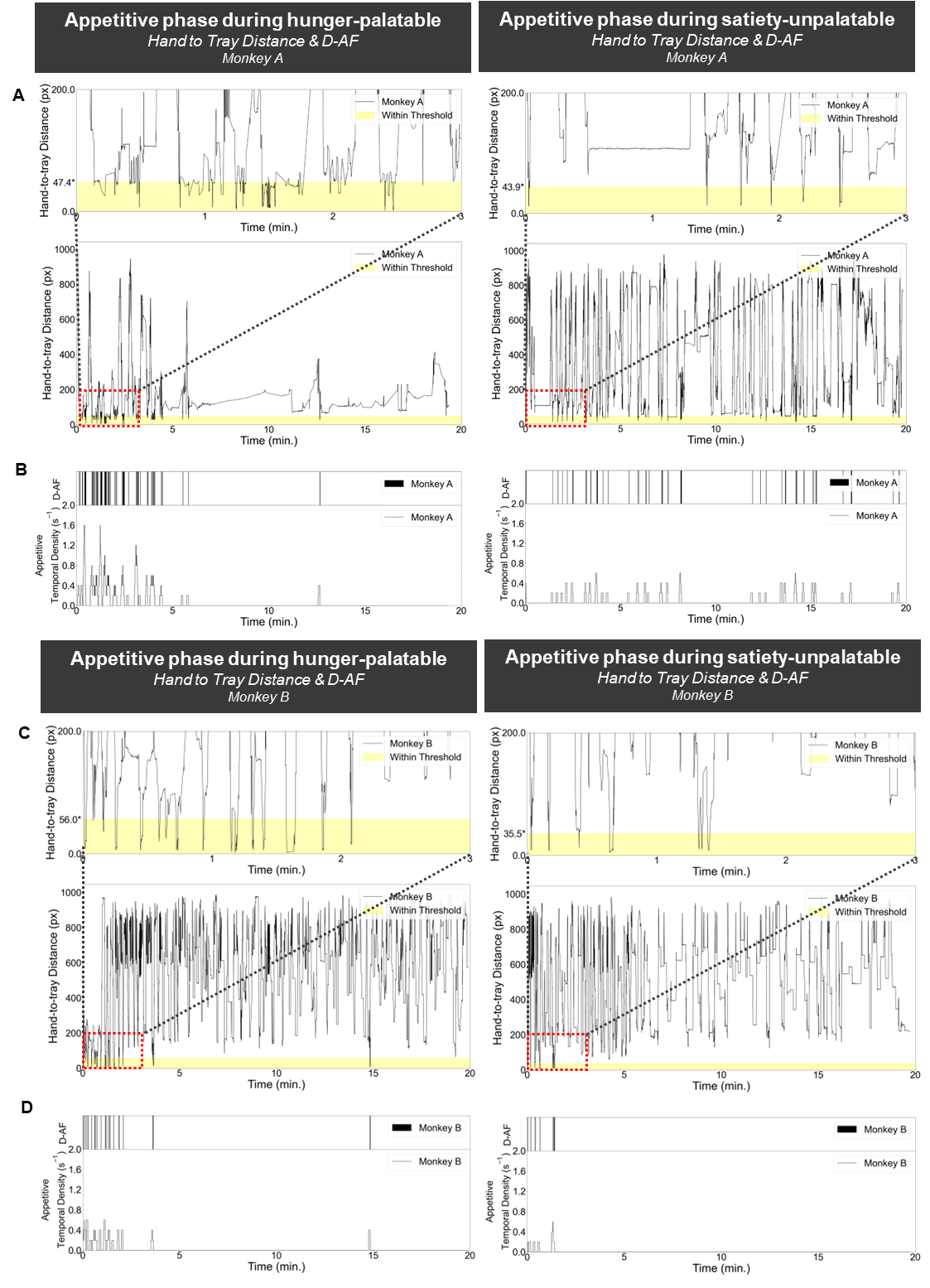
Fig. S7. Temporal dynamics of an eating behavior contributing to D-AF.** **(A)** The minimum of the distances between the tray and right, left hand of Monkey A over time in hunger internal-palatable food (left) and satiety internal-unpalatable food (right) states of artificial food condition 1. For each state, the upper plot shows the first 3 min of the lower plot. The “hands in tray” time was defined by the time below threshold distance (yellow) between the tray and hands. The value with an asterisk (*) is the threshold of distance in each state (see methods; approximately 5.77 px/cm). **(B)** The temporal distribution of D-AF in the same trials as A. Each bar in the upper plot represents moments when a hand approached the tray and the lower plot shows the temporal density of D-AF over time. **(C-D)** The same plots for Monkey B.

**
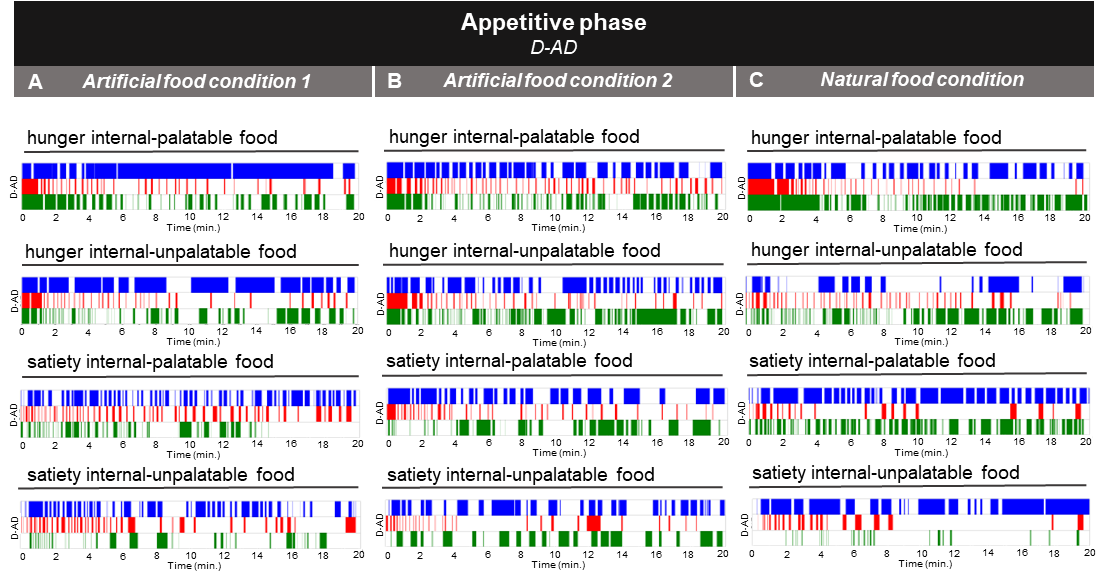
Fig. S8. Temporal dynamics of D-AD.** **(A)** The intervals during which each monkey stayed in the food zone, for Monkeys A, B, and C in hunger internal-palatable food, hunger internal-unpalatable food, satiety-palatable food, and satiety-unpalatable food (from top to bottom) of the artificial food condition1. **(B-C)** The same plots for artificial food condition 2 and natural food condition, respectively.

**
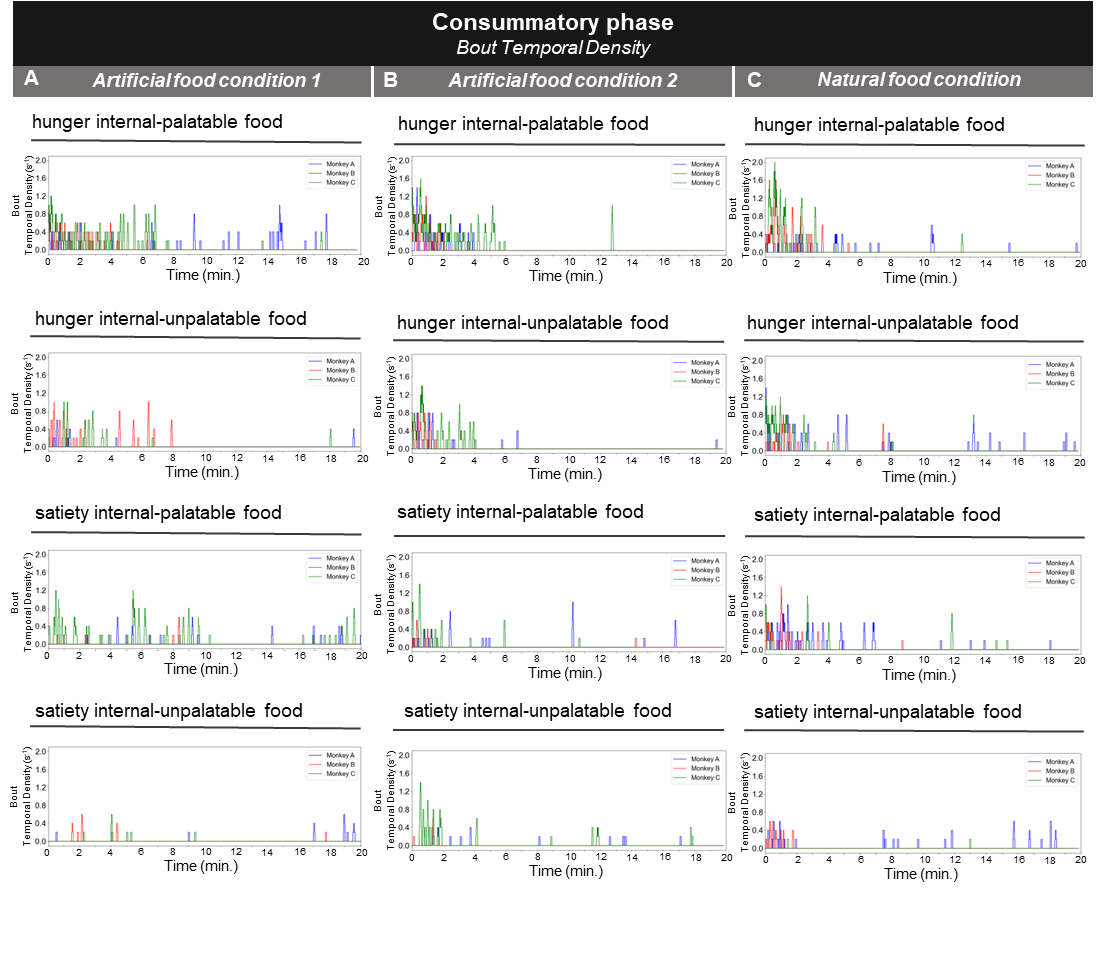
Fig. S9. Temporal dynamics of D-CF.** **(A)** The temporal densities of D-CF of Monkeys A, B, and C in hunger internal-unpalatable food, satiety-palatable food, and satiety-unpalatable food (from top to bottom) of the natural food condition. **(B-C)** The same plots for artificial food condition 1 and artificial food condition 2, respectively.

**
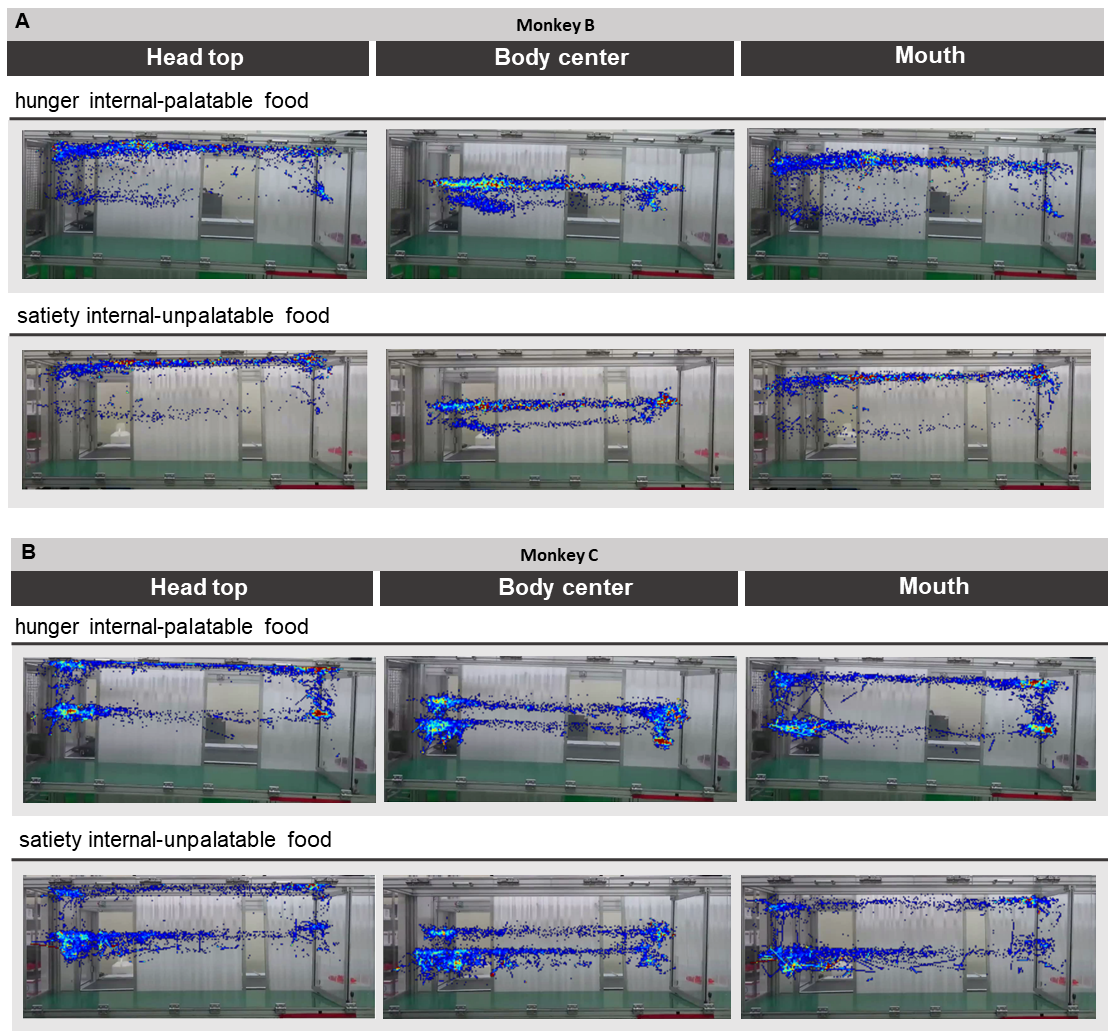
Fig. S10. Positions of the head top, body center, and mouth over time presented as heatmaps.** **(A)** The proportion of time during which the head top (left), body center (middle), and mouth (right) of Monkey B were labelled at each spatial zone over time, in hunger internal-palatable food (upper) and satiety internal-unpalatable food (lower) states of artificial food condition 1. The red region of the heatmaps refers to a higher proportion and the blue region refers to a lower proportion. **(B)** The same plots for the Monkey C.

**
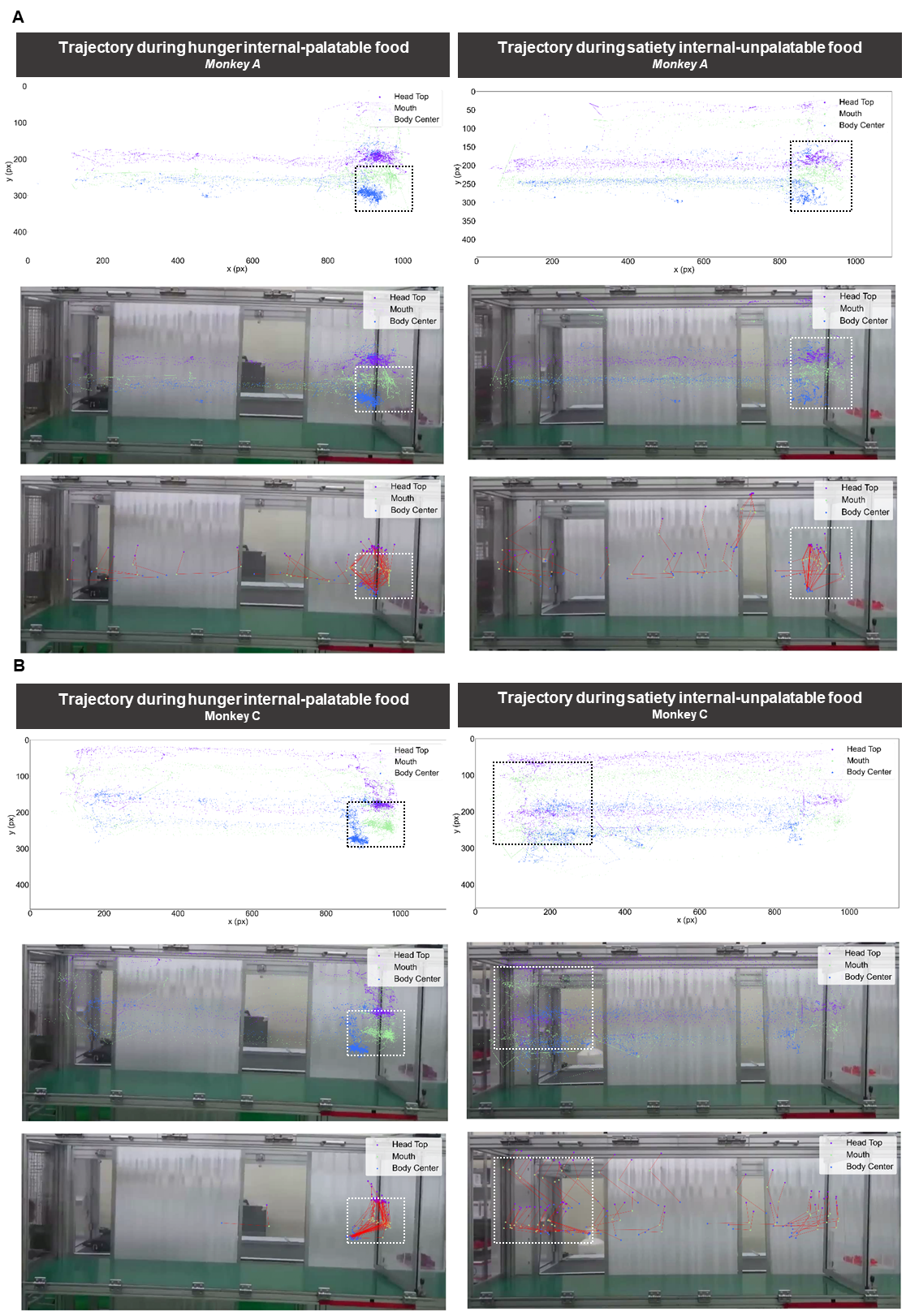
**

**Fig. S11. Positions of the head top, body center, and mouth over time presented as dots and line.** **(A)** The spatial positions of the head top, body center, and mouth of Monkey A in hunger internal-palatable food (left) and satiety internal-unpalatable food (right) states of artificial food condition 1. For each state, the plots at the top and middle present the positions of the body parts at each frame over the whole time as dots, without and with a background image respectively. The plot at the bottom presents the average positions of the body parts for every 1 sec over the first one minute as dots, with lines connecting head top-mouth and mouth-body center. **(B)** The same plots for Monkey C.


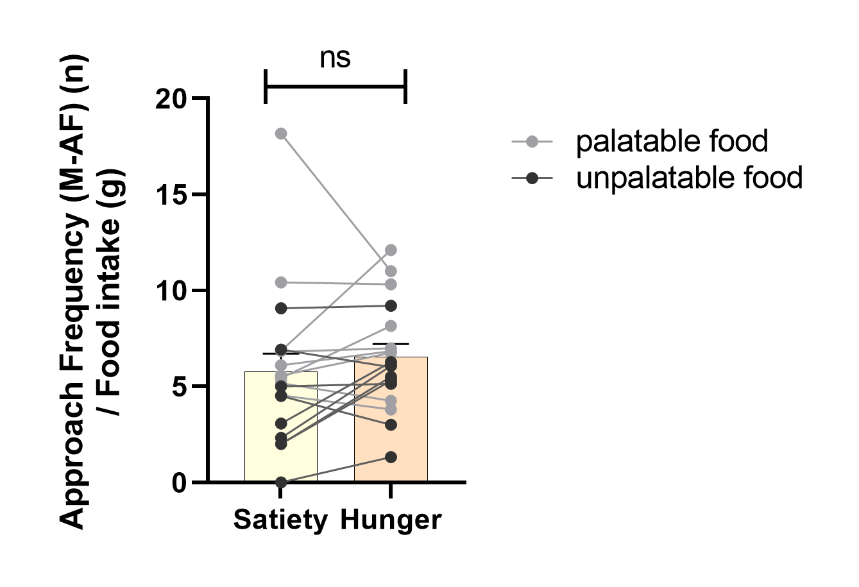
**Fig. S12. Approach Frequency (M-AF) divided by total amount of food intake (g).** There is no significant difference ratio, indicating that the quantity taken by the monkeys at once did not vary based on internal states.

**
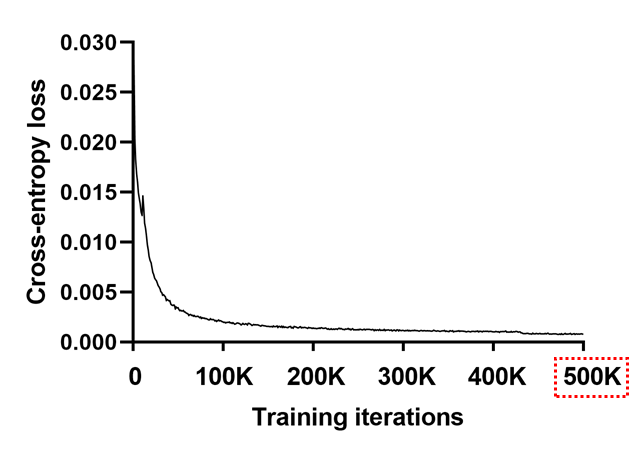
Fig. S13. The value of loss against the number of training iterations.** The loss values of our data converged to values close to 0 after 500,000 training iterations (red dotted line: our training iterations), indicating saturation. The final loss value after running 500K training iterations was 0.00077.

|  | | Unpalatable food | | | Palatable food | | | Satiety internal | | | | Hunger internal | | | |
| --- | --- | --- | --- | --- | --- | --- | --- | --- | --- | --- | --- | --- | --- | --- | --- |
|  | | ***↑↓*** | ***↑↓**** | **sum** | ***↑↓*** | ***↑↓**** | **sum** | ***↑↓*** | ***↑↓**** | | ***sum*** | ***↑↓*** | ***↑↓**** | | **sum** |
| Appetitive | **M-AF** | 2 | 2 | **4** | 1 | 2 | **3** | 2 | | 0 | **2** | 0 | | 0 | **0** |
|  | **M-AD** | 1 | 0 | **1** | 1 | 0 | **1** | 0 | | 0 | **0** | 0 | | 0 | **0** |
|  | **M-AL** | 2 | 0 | **2** | 1 | 0 | **1** | 2 | | 0 | **2** | 2 | | 0 | **2** |
|  | **M-AC** | 2 | 0 | **2** | 3 | 0 | **3** | 2 | | 2 | **4** | 1 | | 0 | **1** |
|  | **D-AF** | 2 | 0 | **2** | 1 | 0 | **1** | 0 | | 0 | **0** | 1 | | 0 | **1** |
|  | **D-AD** | 1 | 0 | **1** | 1 | 0 | **1** | 1 | | 2 | **3** | 2 | | 0 | **2** |
| Consum-  matory | **M-CF** | 0 | 2 | **2** | 1 | 0 | **1** | 3 | | 0 | **3** | 0 | | 2 | **2** |
|  | **M-CD** | 0 | 4 | **4** | 2 | 0 | **2** | 2 | | 2 | **4** | 1 | | 2 | **3** |
|  | **D-CF** | 2 | 0 | **2** | 1 | 2 | **3** | 0 | | 2 | **2** | 1 | | 0 | **1** |

**Table S1. The representative eating behavior indices score with best utility and performance.** 1 point for values in the same direction but statistically non-significant (p-value > 0.05: indicated by black arrow), and 2 points for statistically significant values (p-value < 0.05). For instance, In the context of the appetitive phase behavioral index of M-AF, when considering unpalatable food (first row), we observed that in artificial food condition 1, a statistically significant value was obtained, awarding 2 points. In artificial food condition 2 and natural food, although statistical significance was not achieved, all three monkeys exhibited the same directional trend, each earning 1 point. This cumulative assessment results in a total of 4 points. *↑↓ (p>0.05) : scored for 1*, *↑↓* (p<0.05): scored for 2*

| **Figure 2: Representative feeding behavior indices of the hunger and palatability effect** | | | | | | | | | | | | |
| --- | --- | --- | --- | --- | --- | --- | --- | --- | --- | --- | --- | --- |
| ***Paired***  ***t-test*** | **A. approach frequency** | | | **B. appetitive composite score** | | | **C. consummatory duration** | | | **D. consummatory duration** | | |
| **Between subjects** | **A.F.1** | **A.F.2** | **N.F.** | **A.F.1** | **A.F.2** | **N.F.** | **A.F.1** | **A.F.2** | **N.F.** | **A.F.1** | **A.F.2** | **N.F.** |
|  | 0.0395  * | 0.142 | 0.175 | 0.2365 | 0.0409  * | 0.1835 | 0.0434  * | 0.0438  * | 0.2214 | 0.1707 | 0.0025  ** | 0.0725 |
| **Within subjects** | **Monkey**  **A** | **Monkey**  **B** | **Monkey**  **C** | **Monkey**  **A** | **Monkey**  **B** | **Monkey**  **C** | **Monkey**  **A** | **Monkey**  **B** | **Monkey**  **C** | **Monkey**  **A** | **Monkey**  **B** | **Monkey**  **C** |
|  | 0.1245 | 0.0415  * | 0.1454 | 0.3144 | 0.1809 | 0.0244  * | 0.2186 | 0.0195  * | 0.21 | 0.1668 | 0.0009  *** | 0.2312 |
|  |  |  |  |  |  |  |  |  |  |  |  |  |
| **Figure 4: The consistency between manual and deep learning-based analysis** | | | | | | | | | | | | |
| ***Paired***  ***t-test*** | **A. appetitive duration** | | | **B. deep learning-based appetitive duration** | | | **D. approach frequency** | | | **E. deep learning-based approach frequency** | | |
| **Between subjects** | **A.F.1** | **A.F.2** | **N.F.** | **A.F.1** | **A.F.2** | **N.F.** | **A.F.1** | **A.F.2** | **N.F.** | **A.F.1** | **A.F.2** | **N.F.** |
|  | 0.4775 | 0.082 | 0.9299 | 0.3412 | 0.085 | 0.9945 | 0.0395  * | 0.142 | 0.175 | 0.9704 | 0.1118 | 0.0883 |
| **Within subjects** | **Monkey A** | **Monkey B** | **Monkey C** | **Monkey**  **A** | **Monkey**  **B** | **Monkey**  **C** | **Monkey A** | **Monkey B** | **Monkey C** | **Monkey A** | **Monkey B** | **Monkey C** |
|  | 0.7827 | 0.802 | 0.2044 | 0.9692 | 0.9387 | 0.0858 | 0.1245 | 0.0415  * | 0.1454 | 0.1047 | 0.0255  * | 0.5344 |

**Table S2. The exact *p-value* for each between- and within- subjects of Fig. 2 and Fig. 4**

A.F.1; artificial food condition 1, A.F.2; artificial food condition2, N.F.; natural food condition

* ( *p* < 0.05), ** ( *p* < 0.01), *** ( *p* <0.001)

**Movie S1**. One-food test during the hunger internal-palatable food state measured by manual analysis.

**Movie S2**. One-food test during the satiety internal-unpalatable food state measured by manual analysis.

**Movie S3**. Pairwise artificial food test during the hunger internal state measured by manual analysis.

**Movie S4**. Pairwise natural food test during the hunger internal state measured by manual analysis.

**Movie S5**. Trajectories on hunger internal-palatable food state measured by deep learning-based analysis.

**Movie S6**. Trajectories on satiety internal-unpalatable food state measured by deep learning-based analysis.
